# Exploration of the anti-hyperuricemia effect of TongFengTangSan (TFTS) by UPLC-Q-TOF/MS-based non-targeted metabonomics

**DOI:** 10.1186/s13020-023-00716-w

**Published:** 2023-02-16

**Authors:** Zhichao Huang, Wugang Zhang, Qiong An, Yifan Lang, Ye Liu, Huifang Fan, Haifang Chen

**Affiliations:** 1 Key Laboratory of Modern Preparation of Traditional Chinese Medicine, Ministry of Education, Jiangxi University of Chinese Medicine, 1688 Meiling Road, Nanchang, 330004 China; 2 State Key Laboratory of Innovative Drug and Efficient Energy-Saving Pharmaceutical Equipment, Jiangxi University of Traditional Chinese Medicine, Nanchang, 330006 China

**Keywords:** TFTS, Hyperuricemia, Metabolomics, Mechanism

## Abstract

**Background:**

TongFengTangSan (TFTS) is a commonly used Tibetan prescription for gout treatment. Previously, TFTS (CF) was confirmed to have a significant uric acid-lowering effect. However, the anti-hyperuricemia mechanisms and the main active fractions remain unclear. The current study aimed to investigate the anti-hyperuricemia mechanism using metabolomics and confirm the active CF fraction.

**Methods:**

The hyperuricemia model was established through intraperitoneal injection containing 100 mg/kg potassium oxonate and 150 mg/kg hypoxanthine by gavage. We used serum uric acid (sUA), creatinine (CRE), blood urea nitrogen (BUN), xanthine oxidase (XOD) activity, interleukin-6 (IL-6) and interleukin-1β (IL-1β) as indicators to evaluate the efficacy of CF and the four fractions (SX, CF30, CF60, and CF90). The anti-hyperuricemia mechanism of CF was considered through non-targeted metabolomics depending on the UPLC-Q-TOF–MS technology. Principle component analysis (PCA) and orthogonal partial least squares-discriminant analysis (OPLS-DA) helped explore the potential biomarkers in hyperuricemia. Moreover, the differential metabolites and metabolic pathways regulated by CF and four fractions were also assessed.

**Results:**

CF revealed a significant anti-hyperuricemia effect by down-regulating the level of sUA, sCRE, sIL-1β, and XOD. SX, CF30, CF60, and CF90 differed in the anti-hyperuricemia effect. Only CF60 significantly lowered the sUA level among the four fractions, and it could be the main efficacy fraction of TFTS. Forty-three differential metabolites were identified in hyperuricemia rats from plasma and kidney. Pathway analysis demonstrated that seven pathways were disrupted among hyperuricemia rats. CF reversed 19 metabolites in hyperuricemia rats and exerted an anti-hyperuricemia effect by regulating purine metabolism. CF60 was the main active fraction of TFTS and exerted a similar effect of CF by regulating purine metabolism.

**Conclusions:**

CF and CF60 could exert an anti-hyperuricemia effect by regulating the abnormal purine metabolism because of hyperuricemia while improving intestinal and renal function. CF60 could be the main active fraction of TFTS.

**Supplementary Information:**

The online version contains supplementary material available at 10.1186/s13020-023-00716-w.

## Introduction

Hyperuricemia is a metabolic disease caused by a purine metabolism disorder. It is defined by the serum urate concentration higher than 408 μmoL/L. Continuous high urate levels within the body lead to sodium urate crystal deposition in the joints and eventually causes gout symptoms [1, 2]. Long-term hyperuricemia was associated with chronic kidney disease, hypertension, obesity, and type 2 diabetes [3–6]. Currently, xanthine oxidase inhibitors (allopurinol, febuxostat, and topiroxostat) and uric acid excretion-promoting drugs (probenecid and benzbromarone) are commonly utilized for treating hyperuricemia in the clinic [4, 7]. However, these drugs have adverse reactions, such as diarrhea, abnormal liver function, nausea, headache, etc. [4, 8]. Therefore, safe and effective alternative medicines should be explored.

TongFengTangSan (TFTS) comprises the dried stem of *Tinospora sinensis* (Lour.) Merr. (Le zhe), the dried fruit of *Terminalia chebula* Retz. (He zi), and the dried faces of *Trogopterus xanthipes* Milne-Edwards (Zha xun). We verified the plant names with the Plant List (http://www.theplantlist.org). TFTS was recorded in “The Tibetan Medicine Standard” [9] and “The Great Dictionary of Chinese Medicine” [10]. It is applied to treat gouty diseases in Tibetan and Mongolian areas. As a classic Tibetan prescription, TFTS has been widely utilized in Aba Tibetan hospitals to treat gout and have been confirmed by clinical studies [11, 12]. Our previous study revealed that TFTS possesses the anti-hyperuricemia effect of TFTS (CF) [13]. However, the potential effect and the main fractions of TFTS remain unclear. Recent studies have indicated hydrolyzable tannins, and triterpenes from *Terminalia chebula* revealed good anti-inflammatory activity [14, 15]. This includes 2,3,4,6-pentagalloyl-β-D-glucose, 1,3,6-tri-O-galloyl-β-D-glucose, chebulagic acid punicalagin, 4-O-(3ʹʹ,4ʹʹ-diO-galloyl)-α-L-rhamnosyl-ellagic acid, arjunic acid, and arjunolic acid. Urolithin A, the main component of Zha xun and the final intestinal metabolite of ellagic acid, also had an anti-inflammatory effect [16]. Ethyl gallate and tetrahydropalmatine possessed potential anti-gout effects [17, 18]. However, the single-ingredient efficacies neither represent the anti-gout efficacy of TFTS nor reflect the final effectiveness of TFTS after synergistic and antagonistic interactions between the components. Therefore, the potential anti-gout mechanism of TFTS should be explored.

Metabonomics is a branch of systems biology to determine disease biomarkers through the qualitative and quantitative analysis of small molecular metabolites [19, 20]. These metabolites could be the final products of gene expression, revealing the physiological and pathological changes within the body [21]. Therefore, metabonomics facilitates understanding disease pathogenesis and is often applied to clarify the drug intervention mechanism.

Hence, the current study first investigated the anti-hyperuricemia effect of TFTS and four fractions. Then, we applied non-targeted metabonomics technology based on the UPLC-QTOF–MS technique to identify and analyze the changes in endogenous small biomolecules. Principal component analysis (PCA) and orthogonal partial least squares-discriminant analysis (OPLS-DA) was utilized to screen the significant metabolites among hyperuricemia rats. Finally, the anti-hyperuricemia effect difference between CF and four fractions was analyzed. Consequently, 43 potential metabolites were observed in hyperuricemia rats, including 25 metabolites in plasma and 18 metabolites in the kidney. Plasma and kidney metabonomics indicated that seven metabolic pathways were disturbed. This included purine metabolism, arginine biosynthesis, pyrimidine metabolism, aminoacyl-tRNA biosynthesis, beta-alanine metabolism, arginine and proline metabolism, alanine, aspartate and glutamate metabolism. After the treatment of TFTS, 19 metabolites in the plasma and the kidney were reversed. TFTS could exert an anti-hyperuricemia effect through purine metabolism. Additionally, the metabolic pathways regulated by the 60% ethanol elution fraction of TFTS are close to it and could be the primary active fraction. Therefore, these findings provide a significant basis for the clinical application of TFTS within Tibetan areas.

## Materials and methods

### Reagents

Allopurinol, hypoxanthine, and potassium oxonate were procured from Sigma-Aldrich Corporation Co. Ltd (MO, USA). Uric acid (UA), blood urea nitrogen (BUN), xanthine oxidase (XOD), creatinine (CRE), interleukin-6 (IL-6), and interleukin-1β (IL-1β) assay kit were obtained from Nanjing Jiancheng Bioengineering Institute (China). Carboxymethylcellulose sodium salt (CMC-Na) was purchased from Sinopharm Chemical Reagent Co., Ltd (China). Moreover, UPLC-grade methanol, acetonitrile, and ammonium acetate were supplied by the Tedia Company Inc. (Fairfield, USA). Water was prepared with the ultrapure water purifying system of Hitech Instruments Co., Ltd (China). All the incorporated chemicals were of analytical grade.

### Plant materials and formula compositions

Le ze (No. 20150329), He zi (No. 20150329), and Zha xun (No. 20151104) were purchased from Sichuan Zhongyong Pharmaceutical Co., Ltd. Professor Yanqin Xu (Jiangxi University of Chinese Medicine) identified the raw materials*.*

### Animals and drug administration

Male Sprague–Dawley (SD) rats (180–200 g) were obtained from the Hunan Slaike Jingda Laboratory Animal Co. Ltd (Hunan, China). These rats were housed in cages with free access to food and water under standard laboratory conditions. The conditions were at a light/dark cycle for 12 h and a relative humidity of 55% and 23 ± 1 °C. After 5 days of breeding, the experiment was undertaken. The Guide for the Care and Use of Laboratory Animals of the Jiangxi University of Chinese Medicine approved all the animal experiments (2017–004).

### Preparation of TFTS extract

Le ze, He zi, and Zha xun were mixed at a 5:4:2 ratio and refluxed 2 times for 2 h using 10 times the amount of 90% ethanol. The filtrate was rotary evaporated and freeze-dried to obtain the TFTS (CF) extract with a 13.81% yield. Subsequently, CF was adsorbed with AB-8 macroporous adsorption resin and eluted using water, 30%, 60%, and 90% ethanol to procure the corresponding fractions. The yields from SX (water elution), CF30 (30% ethanol elution), CF60 (60% ethanol elution), and CF90 (90% ethanol elution) were 0.78%, 3.97%, 3.02%, and 0.27%, respectively. The MS chromatograms, primary CF constituents, and each fraction are depicted in Additional files 1 and 2.

### UPLC-Q-TOF–MS analysis

The UPLC-Q-TOF–MS analysis was performed with the Agilent 1290 Infinity (Agilent, USA) ultra-high performance liquid chromatography (UPLC) coupled with Triple Q-TOF 6600 (AB SCIEX, USA) high-resolution mass spectrometry. The electrospray ion source (ESI) parameters were set as follows: source temperature (TEM, 600 °C); ion spray voltage (ISVF, 4 kV); ion source gas1 (GS1, 60 psi), ion source gas 2 (GS2, 60 psi), and curtain gas (CUR): 35 psi. In both positive and negative ionization modes, collision energy (CE) and declustering potential (DP) were set at 30 eV and 60 V. The data were obtained using the information-dependent acquisition (IDA) mode over a mass range between 60 and 1200 m/z.

Chromatographic separation was performed using a Waters ACQUITY UPLC BEH Amide C18 column (2.1 × 100 mm, 1.7 μm, USA). The column temperature was maintained at 25 °C with a flow rate of 0.5 mL/min. The gradient mobile phase had water (containing 25 mmoL/L ammonium acetate and 25 mmoL/L ammonia) (phase A) and acetonitrile (phase B). The gradient program was: 0 ~ 0.5 min, 95% B; 0.5 ~ 7 min, 95% ~ 65% B; 7 ~ 8 min, 65% ~ 40% B; 8 ~ 9 min, 40% B; 9 ~ 9.1 min, 40% ~ 95% B; and 9.1 ~ 12 min, 95% B. The injection volume was 2 μL.

### Preparation of hyperuricemia model rats and drug treatment

The hyperuricemia model was established by administering 150 mg/kg hypoxanthine by gavage and 100 mg/kg potassium oxonate intraperitoneal injection daily. Hypoxanthine and potassium oxonate were all dissolved in 0.5% CMC-Na solution. We randomly divided the hyperuricemia rats into eight groups (n = 10), such as control, model, allopurinol (15 mg/kg), CF (0.29 g/kg), SX (0.0164 g/kg), CF30 (0.0834 g/kg), CF60 (0.0634 g/kg), and CF90 (0.0056 g/kg) groups. Allopurinol is an inhibitor of uric acid production and was selected as the positive drug. According to the CF efficacy experiment on hyperuricemia rats, its anti-hyperuricemia dosage was set with 2.1 g crude drug/kg. The dosage of four fractions of CF was converted according to the yield of each eluent of CF. The control group and model group were given intragastric and intraperitoneal injections of 0.5% CMC-Na solutions. All the other groups were administered the corresponding drugs by gavage 1 h after model establishment and administered continuously for 9 days. The rats were fasted and provided water before the end of the experiment. After 1 h of administering the drugs, blood was collected from the orbital venous plexus of rats and centrifuged at 3000 rpm/min for 10 min at 4 ℃. The upper plasma and serum were collected and stored at − 20 °C for further testing.

### Preparation of biological samples of plasma and kidney metabolomics

Each 100 μL aliquot of the plasma sample was mixed with 400 μL extraction solution (methanol: acetonitrile = 1:1, V/V). It contained an isotope-labeled internal standard mixture and was vortex-mixed for 30 s. Then, the plasma samples were sonicated in the ice water bath for 10 min and kept standing for 1 h at 4 ℃. 25 mg of kidney tissue was added to 500 μL extract solution (acetonitrile: methanol: water = 2: 2: 1) containing isotopically-labelled internal standard mixture. After 30 s vortex, the kidney samples were homogenized at 35 Hz for 4 min and sonicated for 5 min in ice-water bath. The homogenization and sonication cycle was repeated for 2 times. Then the samples were incubated at − 40 ℃ for 1 h. Then, all biological samples centrifuged at 12000 rpm for 15 min at 4 ℃. 400 μL of the supernatant was eluted and dried using a vacuum. Then the residue was resolved in a certain volume 50% acetonitrile and centrifugated at 12000 rpm for 15 min. Finally, a 75 μL supernatant aliquot was used for MS analysis. In addition, an equal aliquot of the supernatants from all of the samples was mixed as quality control (QC) samples.

### Biochemical analysis

Based on the requirements of the test kits, the levels of serum uric acid (sUA), creatinine (CRE), blood urea nitrogen (BUN), serum xanthine oxidase (sXOD), liver xanthine oxidase (lXOD), interleukin-6 (IL-6), and interleukin-1β (IL-1β) were detected.

### Data processing and multivariate analysis

The raw liquid mass data were converted using the ABF converter software (ver. 1.3, Reifycs Inc) and imported within the MS-DIAL software (Ver. 4.80, Riken Center for Sustainable Resource Science). A series of data processing, including peak extraction, peak alignment, peak identification, and peak area normalization, was performed. Then, a comprehensive data matrix, such as M/Z, RT, and normalized data, was generated. SIMCA-P 13.0 software package (Umetrics, Umea, Sweden) was applied for PCA and orthogonal partial least-squares discrimination analysis (OPLS-DA) on the data matrix. PCA is an unsupervised dimensionality reduction method for observing the global clustering trends and dispersion within the groups. OPLS-DA analysis as a supervised modeling method can remove data variables independent of the independent variable X and the categorical variable Y. It can distinguish the different metabolites among the various groups. These potential biomarkers were screened depending on the VIP value > 1 in the OPLS-DA model with a t-test at *p* < 0.05. The metabolites were identified by analyzing the information of precursor and product ions in mass spectrometry, which were confirmed by HMDB (http://www.hmdb.ca/) and MoNA (https://mona.fiehnlab.ucdavis.edu) databases. Pathway analysis of the identified differential metabolites was performed using the online website MetaboAnalyst 5.0 (https://www.metaboanalyst.ca/MetaboAnalyst/home.xhtml). The semi-quantitative statistics for the differential metabolites were performed using the relative peak area, including the relative peak of the model group compared with the control group and the relative peak of the drug group compared with the model group.

### Hematoxylin and Eosin (H & E) staining

Hematoxylin and Eosin (H & E) staining was utilized to examine the histopathology of the kidney. The kidney tissues were embedded in paraffin (MEIKO EC360, Germany) and cut in 4-µm thickness using a rotary microtome (LEICARM2245, Germany), and stained using H&E. The pathological alteration of the kidney was observed under a microscope (OLYMPUS BX43, Japan) at 40X or 200X magnification.

### Statistical analysis

All the data are expressed as mean ± standard deviation, and the statistical analyses were performed using one-way ANOVA and t-tests with GraphPad Prism 9 (San Diego, CA 92,108, USA.). Pearson correlation analysis was performed between the drug efficacy and differential metabolites using SPSS 17.0 (Chicago, IL, USA). *p* < 0.05 was considered statistically significant, and *p* < 0.01 represented highly significant data.

## Results

### Effect of TFTS on sUA, CRE, BUN, XOD, sIL-1β, and sIL-6 within the serum of hyperuricemia rats

**Fig.1:**
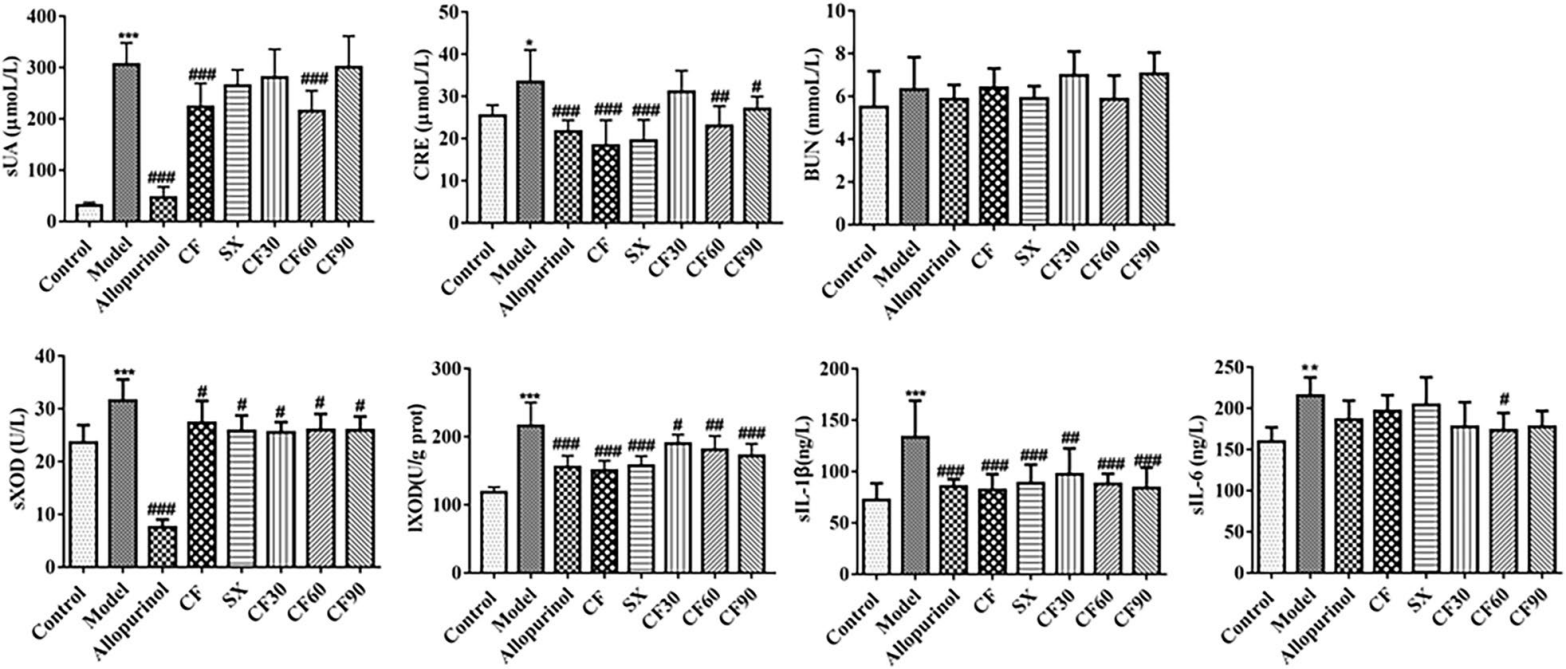
Anti-hyperuricemia effect of CF and four fractions. Compared with control group, *p < 0.05, **p < 0.01, ***p < 0.001; compared with model group, ^#^*p* < 0.05, ^##^*p* < 0.01, ^###^*p* < 0.001

The increased uric acid in the blood has been the main feature of hyperuricemia. As shown in Fig. 1, the sUA level in the model group was significantly increased than the control group (*p* < 0.001). Thus, it demonstrated the successful establishment of the hypoxanthine and potassium oxonate-induced hyperuricemia model. After CF treatment, the sUA level was significantly decreased than the model group (*p* < 0.001. Only CF60 decreased sUA triggered by hyperuricemia among the four fractions. XOD, one of the sources of uric acid production, was significantly elevated in the model group than in the control group. After CF treatment, the level of sXOD and lXOD was significantly lower than that in the model group (*p* < 0.05, *p* < 0.001). The results indicated that treatment with four fractions of CF, such as SX, CF30, CF60, and CF90, reduced the level of sXOD or lXOD than in the model group (*p* < 0.05). The CRE level in hyperuricemia rats was higher than that in the control group (*p* < 0.05). Therefore it depicted the accompanied renal function injury in the hyperuricemia model. After treatment using CF, SX, CF60, and CF90, the CRE level significantly decreased compared to the model group (*p* < 0.05, *p* < 0.01, *p* < 0.001). The levels of sIL-1β and sIL-6 in hyperuricemia rats were also higher than that in the control group (*p* < 0.01, *p* < 0.001), demonstrating the accompanied inflammatory reaction in hyperuricemia rats. After the administration of CF and four fractions, the sIL-1β levels were significantly decreased (*p* < 0.01, *p* < 0.001). Among four fractions, only CF60 significantly reduced the level of sIL-6 (*p* < 0.05).  In this experiment, no significant difference was observed on the BUN level of hyperuricemia rats.  In general, only CF and CF60 exerted the down-regulation effect on uric acid, the final indicator of hyperuricemia. Therefore, it can be speculated that CF60 could be the main fraction of TFTS.

### TFTS ameliorated Histopathological changes in kidney

**Fig.2:**
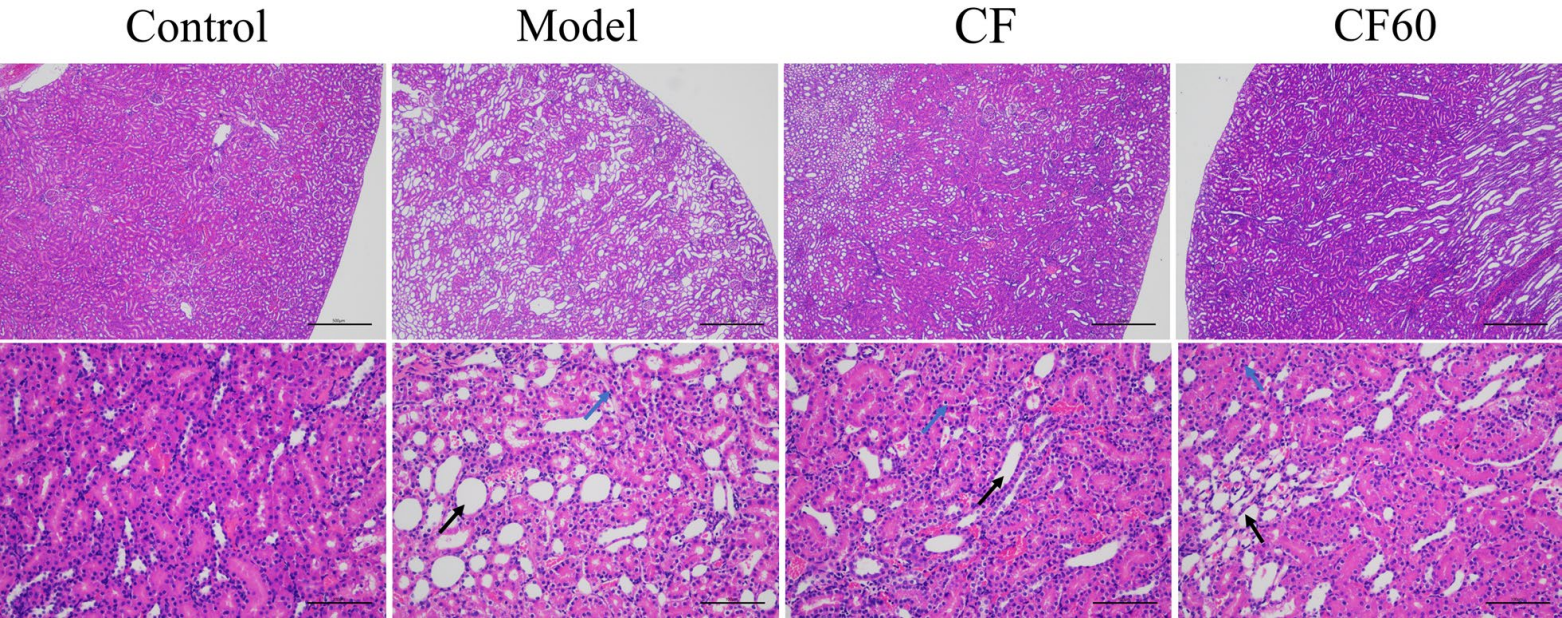
Histopathological study of kidney (× 40 and × 200).The renal tubules in kidney exhibited expansion (black arrow) and vacuolar degeneration (blue arrow)

We evaluated the therapeutic effect by analyzing the results of HE stains of kidney tissue to verify the improvement of the pathological state of the kidney in hyperuricemia rats after administering CF and CF60. Kidney histological changes in HX and PO-induced hyperuricemic rats are demonstrated in Fig. 2. In the model group, the renal tubules in the renal cortex expanded in a large area under a 40-fold microscope. The expansion degree and area of renal tubules in the renal cortex were significantly improved after the administration of CF and CF60. At 200 fold microscope, the epithelial cells of renal tubules were flat,and renal tubules were dilated and vacuolated inside the model group. After administering CF and CF60, the expansion degree and vacuolar degeneration of renal tubules are improved. These results demonstrate that CF and CF60 had the potential anti-hyperuricemia effect, establishing that CF60 was the main TFTS fraction.

### Establishment of plasma and kidney metabolism atlas

**Fig.3:**
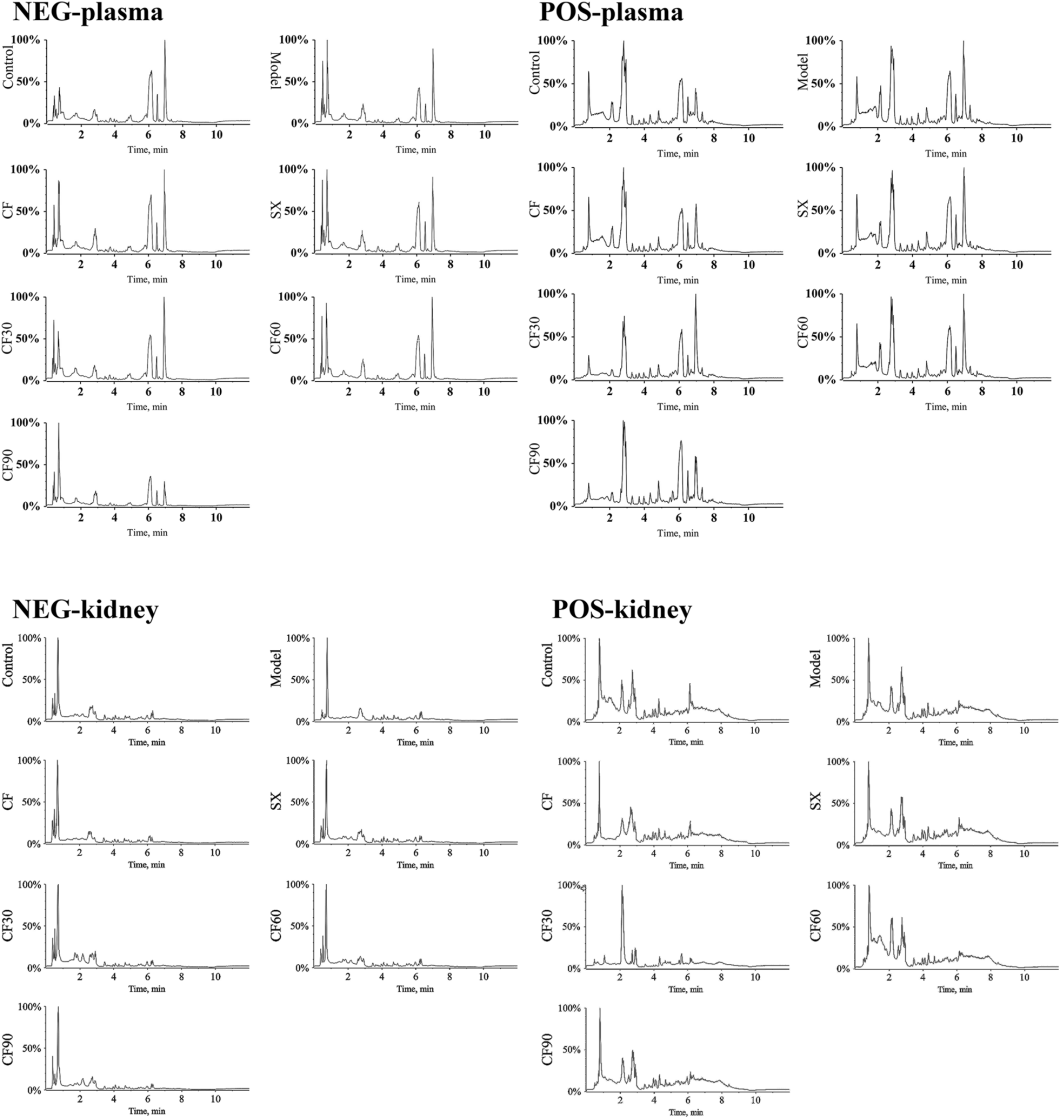
TIC of differential metabolites in plasma and kidney samples obtained in positive and negative modes

UPLC-Q-TOF–MS methods detected the plasma and kidney samples, and identified the endogenous molecules in positive and negative modes. TIC chromatograms of biological samples from TFTS, SX, CF30, CF60, and CF90 are represented in Fig. 3. To evaluate data quality and reliability, all QC sample data was analyzed. The peak area relative standard deviations of internal standard were within the range of 1.47–4.46% (Additional file 3). As shown in Additional file 4, the peak area deviations of all QC samples obtained by PCA were less than 2 SD. This result indicated that the UPLC-MS/MS system was stable and the data was reliable.

### PCA analysis and OPLS-DA analysis

**Fig.4:**
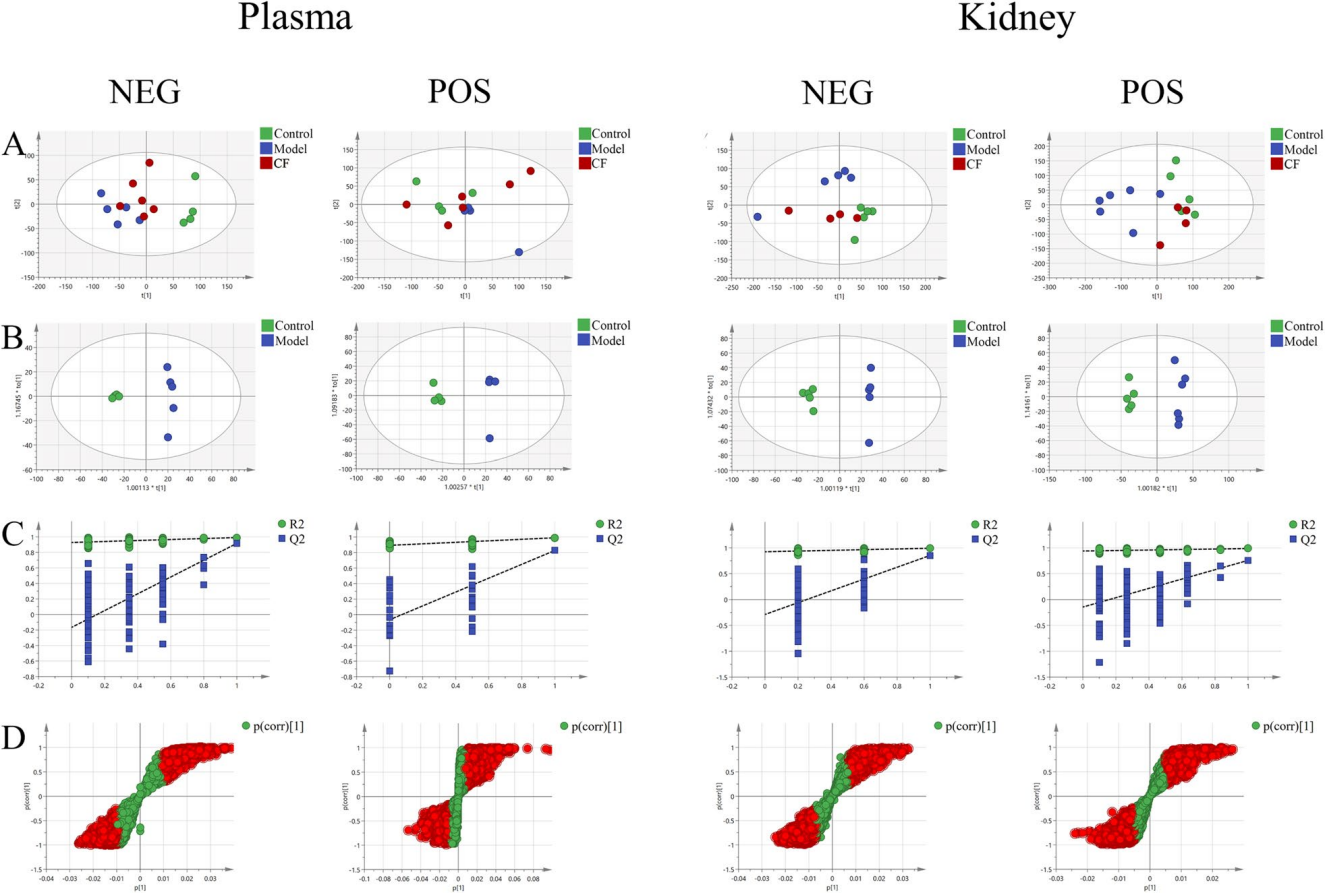
Multivariate statistical analysis of metabolomics. **A** PCA score plot. **B** OPLS-DA score plot. **C** Permutation Plot. **D** S-plot

PCA model and an OPLS-DA model were established in both the positive and negative ion modes to obtain metabolic characteristics between the control and model groups. As shown in Fig. 4A, the metabolome profiles between the control and model groups were separated into two clusters in negative mode and that of the CF group was between the model and the control groups. Therefore, the endogenous metabolites changed under the pathological state and were normal after CF treatment. In the positive ion mode, the metabolome profiles of control and model groups could also be separated to a certain extent. However, the separation degree was not as good as in the negative ion mode. After the CF intervention, the endogenous metabolites of the CF group were also closer to the control group. The OPLS-DA model was built to screen the different metabolites between the control and model groups. Figure 4B showed an evident separation between the control and model groups. Thus, the corresponding permutation test results show that the models are not overfitting (Fig. 4C), indicating that the OPLS-DA model is reliable with good applicability and predictability. Differential metabolites between the control and model groups are shown in Fig. 4D. The red dots in the figure indicate VIP values of substances greater than 1. The above results showed that the OPLS-DA model possesses good prediction ability.

### Potential biomarker analysis

**Fig.5:**
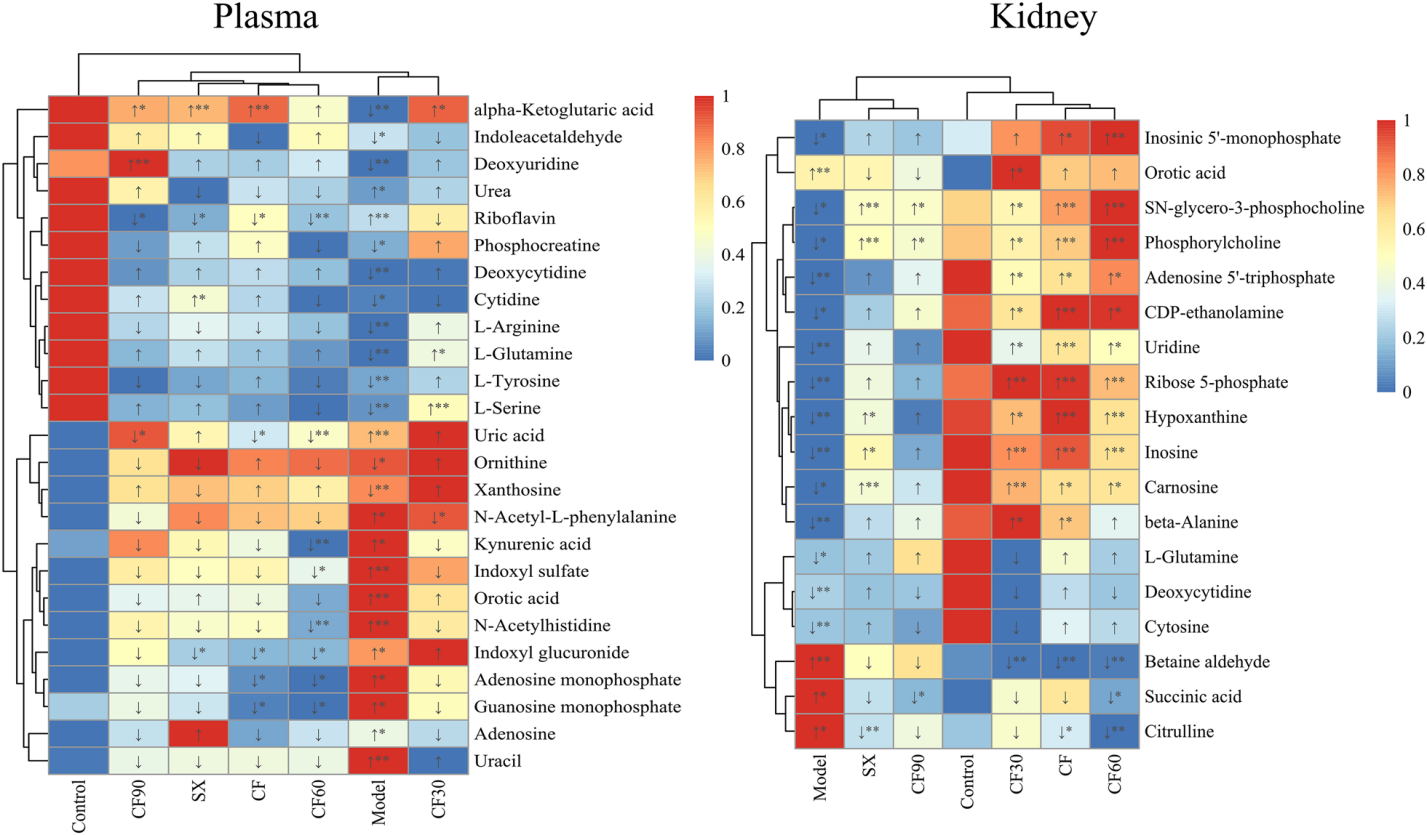
Heatmap of the differential metabolites between CF and four fractions. *p < 0.05, **p < 0.01. Red: up-regulation; Blue: down-regulation

OPLS-DA model combined with a t-test was applied to analyze and screen the biomarkers between the control and model groups in plasma. These biomarkers were introduced into HMDB and MoNA databases for identification. Forty-three potential biomarkers from plasma and kidney were identified, as indicated in Table 1 and Fig. 5. These potential biomarkers were screened based on the VIP value > 1 in the OPLS-DA model and* p* < 0.05 by t-test. Six of the 25 differential plasma metabolites were significantly reversed after CF treatment (*p* < 0.05). This included alpha-ketoglutaric acid, guanosine monophosphate (GMP), indoxyl glucuronide, uric acid, riboflavin, and adenosine monophosphate (AMP). The regulation effect of four fractions on the metabolites was also investigated. SX group significantly reversed the four differential plasma metabolites (riboflavin, indoxyl glucuronide, alpha-ketoglutaric acid, and cytidine) (*p* < 0.05, *p* < 0.01) than the model group. CF30 group significantly reversed four differential plasma metabolites compared with the model group. The metabolites were N-acetyl-L-phenylalanine, L-glutamine, L-serine, and alpha-ketoglutaric acid (*p* < 0.05, *p* < 0.01). CF60 group significantly reversed eight differential plasma metabolites (*p* < 0.05, *p* < 0.01): AMP, kynurenic acid, uric acid, GMP, riboflavin, N-acetylhistidine, indoxyl sulfate, and indoxyl glucuronide. CF90 group significantly reversed four different plasma metabolites (*p* < 0.05,* p* < 0.01): uric acid, alpha-ketoglutaric acid, deoxyuridine, and riboflavin.

**Table 1:** Differential metabolites in the plasma and the kidney of hyperuricemia rats

Metabolite name	Formula	Average Rt(min)	Average Mz	Adduct type	HMDB ID	Reference value	ppm	MS^2^	Sample	VIP value	P value
Deoxyuridine	C_9_H_12_N_2_O_5_	1.864	227.0676	[M-H]^−^	0000012	227.0674	1.0	111.0202,41.998	Plasma	1.27	0.0402
Deoxycytidine	C_9_H_13_N_3_O_4_	3.302	226.0854	[M-H]^−^	0000014	226.0833	4.6	135.0572,93.0476,66.0336,41.9999	Plasma	1.07	0.0038
Adenosine monophosphate	C_10_H_14_N_5_O_7_P	6.817	348.0689	[M + H]^+^	0000045	348.0704	− 4.3	136.0601,97.0269	Plasma	1.68	0.0409
Adenosine	C_10_H_13_N_5_O_4_	2.711	268.0995	[M + H]^+^	0000050	268.104	1.6	136.0609,119.0304,94.039	Plasma	1.90	0.0107
Cytidine	C_9_H_13_N_3_O_5_	3.858	242.0749	[M-H]^−^	0000089	242.0782	− 1.4	109.038,91.0302,81.0436,67.027, 41.9966	Plasma	1.04	0.0153
L-Tyrosine	C_9_H_11_NO_3_	4.679	180.0656	[M-H]^−^	0000158	180.0666	− 5.5	180.0605,163.0388,133.0532,119.0486, 107.0493,93.0347,72.0086	Plasma	1.15	0.0012
L-Serine	C_3_H_7_NO_3_	5.926	104.0353	[M-H]^−^	0000187	104.0353	0.1	104.0372,74.0231,72.0069	Plasma	1.19	0.0193
alpha-Ketoglutaric acid	C_5_H_6_O_5_	6.925	145.0117	[M-H]^−^	0000208	145.0143	− 5.9	73.0277,56.9944	Plasma	1.31	0.0080
Ornithine	C_5_H_12_N_2_O_2_	8.17	131.0839	[M-H]^−^	0000214	131.0826	5.6	131.0796,83.0612,68.9961,54.6111	Plasma	2.18	0.0198
Orotic acid	C_5_H_4_N_2_O_4_	3.713	155.0088	[M-H]^−^	0000226	155.0098	− 6.7	111.0194,67.0296,41.999	Plasma	1.38	0.0073
Riboflavin	C_17_H_20_N_4_O_6_	3.347	377.1419	[M + H]^+^	0000244	377.1456	− 4.3	377.1406,243.0913,198.0713	Plasma	1.10	0.0196
Uric acid	C_5_H_4_N_4_O_3_	5.102	167.0227	[M-H]^−^	0000289	167.0211	6.0	167.0217,124.0156,96.0203,69.0089, 41.9989	Plasma	1.11	0.0014
Urea	CH_4_N_2_O	1.718	61.03897	[M + H]^+^	0000294	61.03964	5.4	61.0379,44.016	Plasma	1.23	0.0086
Xanthosine	C_10_H_12_N_4_O_6_	4.881	283.0655	[M-H]^−^	0000299	283.0684	0.2	283.258,151.0266,108.0199	Plasma	2.13	0.0425
Uracil	C_4_H_4_N_2_O_2_	4.879	111.0195	[M-H]^−^	0000300	111.02	− 4.6	68.0191,67.0167,41.9985	Plasma	1.36	0.0002
N-Acetyl-L-phenylalanine	C_11_H_13_NO_3_	2.95	206.0792	[M-H]^−^	0000512	206.0823	− 5.2	164.0695,147.0399,103.0551,91.0539, 72.0083,58.0275	Plasma	2.41	0.0332
L-Arginine	C_6_H_14_N_4_O_2_	8.356	173.1041	[M-H]^−^	0000517	173.1044	− 1.8	131.0795,114.054	Plasma	1.08	0.0033
L-Glutamine	C_5_H_10_N_2_O_3_	5.869	145.0613	[M-H]^−^	0000641	145.0619	− 3.9	109.0394,101.0706,84.0415,74.0238, 58.0279,41.9976	Plasma	1.12	0.0027
Indoxyl sulfate	C_8_H_7_NO_4_S	0.427	212.0208	[M-H]^−^	0000682	212.0023	6.9	211.9986,132.0423,80.9623,79.9613	Plasma	1.64	0.0040
Kynurenic acid	C_10_H_7_NO_3_	3.135	190.0493	[M + H]^+^	0000715	190.0499	− 3.1	162.046,144.0444,116.051,89.0388	Plasma	1.55	0.0165
Indoleacetaldehyde	C_10_H_9_NO	0.534	158.0612	[M-H]^−^	0001190	158.0611	0.1	156.0444,130.0671,129.0593,128.0506	Plasma	1.03	0.0013
Guanosine monophosphate	C_10_H_14_N_5_O_8_P	7.338	364.062	[M + H]^+^	0001397	364.0653	− 4.9	364.141,152.0562	Plasma	1.52	0.0000
Phosphocreatine	C_4_H_10_N_3_O_5_P	6.885	210.026	[M-H]^−^	0001511	210.0285	2.4	969.9699,78.958,55.2934	Plasma	1.08	0.0005
Indoxyl glucuronide	C_14_H_15_NO_7_	4.047	308.0793	[M-H]^−^	0010319	308.0776	5.6	132.0394,75.0004,59.0116	Plasma	1.32	0.0003
N-Acetylhistidine	C_8_H_11_N_3_O_3_	4.908	198.085	[M + H]^+^	0032055	198.0873	3.4	156.0748,110.071,95.059,93.042, 83.0596	Plasma	1.48	0.0003
Deoxycytidine	C_9_H_13_N_3_O_4_	3.289	226.0828	[M-H]^−^	0000014	226.0833	− 2.4	135.0525,93.0422,66.0312,41.997	Kidney	1.53	0.0009
Carnosine	C_9_H_14_N_4_O_3_	5.626	225.1003	[M-H]^−^	0000033	225.0993	4.3	154.0605,137.0329,127.048,110.0698, 93.0439,87.054,81.043	Kidney	1.76	0.0112
beta-Alanine	C_3_H_7_NO_2_	1.441	90.05433	[M + H]^+^	0000056	90.05496	− 7.0	88.0353,59.0111,41.0011	Kidney	1.74	0.0020
SN-glycero-3-phosphocholine	C_8_H_20_NO_6_P	6.156	258.1086	[M + H]^+^	0000086	258.1101	− 5.8	184.0739,166.061,124.9986,104.1068, 86.0966,60.0796	Kidney	2.26	0.0219
Hypoxanthine	C_5_H_4_N_4_O	3.468	137.0454	[M + H]^+^	0000157	137.0458	− 3.1	137.0429,1139.0344,110.0335,94.0377,82.0391,67.0286,55.0296	Kidney	1.86	0.0000
Inosinic acid	C_10_H_13_N_4_O_8_P	7.103	347.0391	[M-H]^−^	0000175	347.0398	− 2.2	347.0296,211.0993,135.0252,96.9625, 78.9559	Kidney	1.28	0.0474
Inosine	C_10_H_12_N_4_O_5_	3.472	267.0738	[M-H]^−^	0000195	267.0735	1.0	135.0271,108.0153,92.0203	Kidney	1.93	0.0000
Orotic acid	C_5_H_4_N_2_O_4_	3.645	155.0094	[M-H]^−^	0000226	155.0098	− 2.9	111.0177,67.0288,59.0111,41.9969	Kidney	3.03	0.0018
Succinic acid	C_4_H_6_O_4_	5.005	117.0198	[M-H]^−^	0000254	117.0193	4.1	99.9228,83.9312	Kidney	1.14	0.0278
Uridine	C_9_H_12_N_2_O_6_	2.6	243.0637	[M-H]^−^	0000296	243.0623	6.0	152.0356,140.0357,122.0251,110.0243,82.0291,66.0337,41.9989	Kidney	1.68	0.0001
Adenosine 5ʹ-triphosphate	C_10_H_16_N_5_O_13_P_3_	7.632	505.9875	[M-H]^−^	0000538	505.9885	− 1.9	505.9939,487.9698,426.0208,408.0068,176.9354,158.9206,78.951	Kidney	1.62	0.0001
Cytosine	C_4_H_5_N_3_O	3.294	112.0497	[M + H]^+^	0000630	112.0505	− 7.9	112.0502,95.0227,94.0403,69.043, 68.1057,67.0284,52.0193,42.0341	Kidney	1.58	0.0001
L-Glutamine	C_5_H_10_N_2_O_3_	5.876	145.0629	[M-H]^−^	0000641	145.0619	7.3	127.0467,109.0358,84.0407,74.0205, 58.0259,41.9958	Kidney	1.01	0.0330
Citrulline	C_6_H_13_N_3_O_3_	6.188	176.1024	[M + H]^+^	0000904	176.103	− 3.5	159.0714,113.0708,70.0646,43.054	Kidney	1.36	0.0139
Betaine-Aldehyde	[C_5_H_12_NO]^+^	6.483	102.0906	[M]^+^	0001252	102.0913	− 7.5	102.0922,59.0741,58.0653	Kidney	2.20	0.0050
Ribose 5-phosphate	C_5_H_11_O_8_P	6.994	229.0129	[M-H]^−^	0001548	229.0119	4.3	96.9659,78.9566	Kidney	1.86	0.0001
CDP-ethanolamine	C_11_H_20_N_4_O_11_P_2_	7.024	445.052	[M-H]^−^	0001564	445.0531	− 2.5	445.0419,383.9898,322.0378,272.9579, 201.9624,96.9638,78.9547	Kidney	1.82	0.0139
Phosphorylcholine	C_5_H_14_NO_4_P	6.155	184.0728	[M + H]^+^	0001565	184.0733	− 2.6	184.0766,124.9976,98.9849,86.0973, 71.0766,60.0826,45.0332	Kidney	2.14	0.0296

Among the 18 differential metabolites from the kidney, 13 were significantly reversed after CF treatment (*p* < 0.05). They were carnosine, beta-alanine, SN-glycero-3-phosphocholine, hypoxanthine, inosine 5'-monophosphate, inosine, uridine, adenosine 5'-triphosphate, citrulline, betaine-aldehyde, ribose 5-phosphate, CDP-ethanolamine, and phosphorylcholine (*p* < 0.05, *p* < 0.01). SX group significantly reversed the six differential metabolites (*p* < 0.05, *p* < 0.01), including carnosine, SN-glycero-3-phosphocholine, hypoxanthine, citrulline, inosine, and phosphorylcholine. CF30 significantly reversed 12 differential metabolites: carnosine, beta-alanine, SN-glycero-3-phosphocholine, hypoxanthine, inosine, orotic acid, uridine, adenosine 5ʹ -triphosphate, betaine-aldehyde, ribose 5-Phosphate, CDP-ethanolamine, and phosphorylcholine. CF60 significantly reversed 13 differential metabolites: carnosine, SN-glycero-3-phosphocholine, hypoxanthine, inosine 5ʹ-monophosphate, inosine, succinic acid, uridine, adenosine 5ʹ-triphosphate, citrulline, betaine-aldehyde, ribose 5-phosphate, CDP-ethanolamine, and phosphorylcholine (*p* < 0.05, *p* < 0.01). Additionally, CF90 significantly reversed three differential metabolites: SN-glycero-3-phosphocholine, succinic acid, and phosphorylcholine (*p* < 0.05).

**Fig.6:**
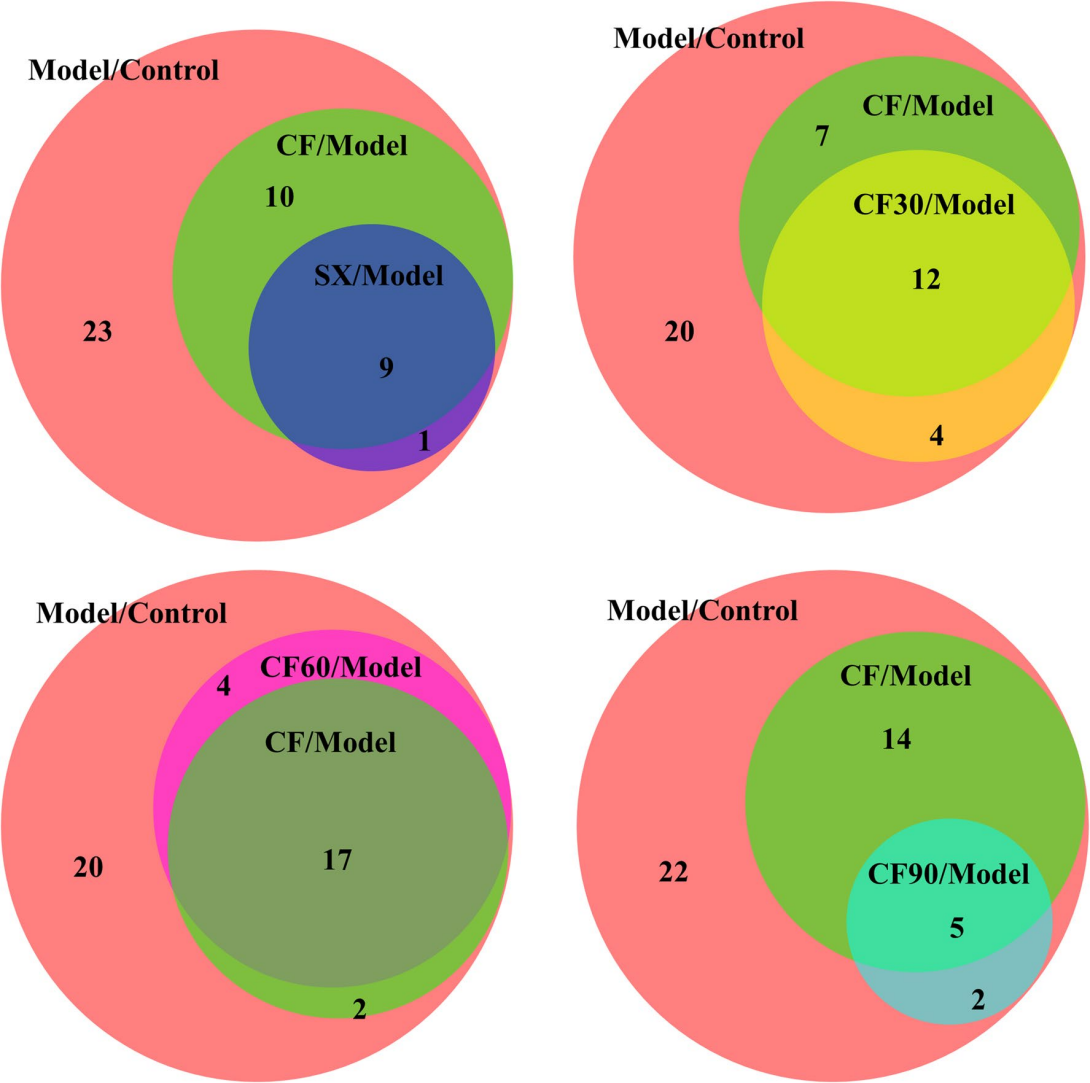
Overlapping metabolites between CF and four fractions illustrated by Venn diagram. Model group vs. Normal group (Model/Control), CF group vs. Model group (CF/Model), SX group vs. Model group (SX/Model), CF30 group vs. Model group (CF30/Model), CF60 group vs. Model group (CF60/Model), CF90 group vs. Model group (CF90/Model)

Forty-three differential metabolites from plasma and kidney were generally disturbed in hyperuricemia rats and listed in Table 2. The differential metabolites among different groups are shown in the Venn diagram (Fig. 6). CF reversed 19 metabolites in hyperuricemia rats. CF60, the main active TFTS fraction, reversed 21 differential metabolites. There are 17 overlapping metabolites between the CF60 group and the CF group. They were significantly more than those between different groups and the CF group. Thus, AMP, GMP, ATP, riboflavin, uric acid, indoxyl glucuronide, carnosine, SN-glycero-3-phosphocholine, hypoxanthine, inosine 5'-monophosphate, inosine, uridine, citrulline, betaine-aldehyde, ribose 5-phosphate, CDP-ethanolamine, and phosphorylcholine could be the potential biomarkers of TFTS treatment for hyperuricemia.

**Table 2:** Semi-quantitative statistics for the differential metabolites regulated by CF and four fractions

Metabolite name	Sample	Model/Control	CF/Model	SX/Model	CF30/Model	CF60/Model	CF90/Model	Allopurinol/Model	Pathway
Deoxyuridine	Plasma	0.5↓^**^	1.3↑	1.2↑	1.2↑	1.3↑	2.1↑^**^	1.2↑	c
Deoxycytidine	Plasma	0.6↓^**^	1.2↑	1.1↑	1↑	1.1↑	1↑	1.2↑	c
Adenosine monophosphate	Plasma	1.9↑^*^	0.6↓^*^	0.7↓	0.8↓	0.5↓^*^	0.7↓	0.7↓	a
Adenosine	Plasma	2.6↑^*^	0.6↓	1.9↑	0.8↓	0.8↓	0.8↓	1.6↑	a
Cytidine	Plasma	0.6↓^*^	1.1↑	1.3↑^*^	1↓	1↓	1.2↑	1.2↑	c
L-Tyrosine	Plasma	0.6↓^**^	1↑	1↓	1.1↑	0.9↓	0.9↓	1↓	d
L-Serine	Plasma	0.6↓^**^	1↑	1.1↑	1.3↑^**^	0.9↓	1.1↑	1↑	d
alpha-Ketoglutaric acid	Plasma	0.5↓^**^	1.8↑^**^	1.7↑^**^	1.8↑^*^	1.4↑	1.7↑^*^	0.8↓	b
Ornithine	Plasma	0.6↓^*^	1↑	1↓	1.2↑	0.9↓	1↓	1↑	b,e
Orotic acid	Plasma	5.3↑^**^	0.9↓	1.1↑	1.1↑	1↓	0.8↓	0.8↓^*^	c
Riboflavin	Plasma	1.5↑^**^	0.8↓^*^	0.8↓^*^	0.9↓	0.7↓^**^	0.8↓^*^	0.8↓	f
Uric acid	Plasma	4.3↑^**^	0.8↓^*^	1↑	1.1↑	0.7↓^**^	0.6↓^*^	0.2↓^**^	a
Urea	Plasma	1.3↑^*^	0.8↓	0.9↓	1.1↑	0.9↓	1.1↑	1↓	a,b
Xanthosine	Plasma	0.5↓^**^	1.2↑	0.9↓	1.1↑	1.1↑	1.4↑	3.1↑^**^	a
Uracil	Plasma	5↑^**^	0.9↓	0.9↓	1.2↑	0.7↓	0.8↓	0.6↓^*^	c
N-Acetyl-L-phenylalanine	Plasma	2.3↑^*^	0.7↓	0.7↓	0.4↓^*^	0.7↓	0.7↓	1.3↑	
L-Arginine	Plasma	0.6↓^**^	1↓	1↓	1.2↑	0.9↓	1↓	1↓	b,d,e
L-Glutamine	Plasma	0.6↓^**^	1.1↑	1.2↑	1.2↑^*^	1.1↑	1.1↑	1.1↑	a,b,c,d,g
Indoxyl sulfate	Plasma	2.8↑^**^	0.7↓	0.7↓	0.9↓	0.6↓^*^	0.7↓	0.8↓	
Kynurenic acid	Plasma	1.8↑^*^	0.7↓	0.8↓	0.8↓	0.5↓^**^	0.9↓	0.6↓^*^	
Indoleacetaldehyde	Plasma	0.6↓^*^	0.8↓	1.2↑	0.9↓	1.2↑	1.3↑	1.3↑	
Guanosine monophosphate	Plasma	1.7↑^*^	0.5↓^*^	0.6↓	0.7↓	0.5↓^*^	0.7↓	1↓	a
Phosphocreatine	Plasma	0.6↓^*^	1.3↑	1.1↑	1.5↑	0.9↓	1↓	0.9↓	e, g
Indoxyl glucuronide	Plasma	2.1↑^*^	0.6↓^*^	0.6↓^*^	1.1↑	0.6↓^*^	0.8↓	0.7↓	i
N-Acetylhistidine	Plasma	1.6↑^**^	0.8↓	0.8↓	0.8↓	0.7↓^**^	0.8↓	0.8↓	
Deoxycytidine	Kidney	0.6↓^**^	1↑	1↑	0.8↓	1↓	1↓	1.3↑	c
Carnosine	Kidney	0.5↓^*^	1.7↑^*^	1.5↑^**^	1.8↑^**^	1.6↑^*^	1.3↑	1.2↑	h
β-Alanine	Kidney	0.6↓^**^	1.5↑^*^	1.3↑	1.7↑^*^	1.3↑	1.3↑	1.2↑	c,h
SN-glycero-3-phosphocholine	Kidney	0.4↓^*^	3.1↑^**^	2.3↑^**^	2.4↑^*^	3.6↑^**^	2.2↑^*^	1.3↑	
Hypoxanthine	Kidney	0.6↓^**^	1.8↑^**^	1.5↑^*^	1.6↑^*^	1.5↑^**^	1↑	1.5↑^*^	a
Inosine 5ʹ- monophosphate	Kidney	0.6↓^*^	2.6↑^*^	1.8↑	2.4↑	2.7↑^**^	1.3↑	1.3↑	a
Inosine	Kidney	0.5↓^**^	2↑^**^	1.7↑^*^	1.9↑^**^	1.7↑^**^	1.1↑	2.1↑^**^	a
Orotic acid	Kidney	6.4↑^**^	1.2↑	0.9↓	1.6↑^*^	1.3↑	0.8↓	0.7↓	c
Succinic acid	Kidney	1.4↑^*^	0.9↓	0.8↓	0.8↓	0.8↓^*^	0.8↓^*^	0.8↓	
Uridine	Kidney	0.6↓^**^	1.5↑^**^	1.4↑	1.3↑^*^	1.4↑^*^	1↑	1.2↑	c
Adenosine 5ʹ-triphosphate	Kidney	0.6↓^**^	1.5↑^*^	1.1↑	1.4↑^*^	1.6↑^*^	1.2↑	1↓	a
Cytosine	Kidney	0.7↓^**^	1.1↑	1.1↑	0.9↓	1↑	0.9↓	1.1↑	c
L-Glutamine	Kidney	0.7↓^*^	1.1↑	1↑	0.9↓	1↑	1.2↑	1↑	a, c, e
Citrulline	Kidney	1.4↑^*^	0.7↓^*^	0.7↓^**^	0.8↓	0.6↓^**^	0.8↓	0.8↓	e
Betaine-Aldehyde	Kidney	2.4↑^**^	0.4↓^**^	0.7↓	0.4↓^**^	0.4↓^**^	0.8↓	0.4↓^**^	
Ribose 5-phosphate	Kidney	0.5↓^**^	2.1↑^**^	1.7↑	2.1↑^**^	1.8↑^**^	1.2↑	1.5↑^*^	a
CDP-ethanolamine	Kidney	0.5↓^*^	2.3↑^**^	1.4↑	1.9↑^*^	2.3↑^*^	1.6↑	1↑	
Phosphorylcholine	Kidney	0.4↓^*^	2.7↑^**^	2.4↑^**^	2.4↑^*^	3.5↑^**^	2.1↑^*^	1.3↑	

a: purine metabolism

b: Arginine biosynthesis

c: Pyrimidine metabolism

d: Aminoacyl-tRNA biosynthesis

e: Arginine and proline metabolism

f: Riboflavin metabolism

g: Alanine, aspartate and glutamate metabolism

H: beta-Alanine metabolism

I: Pentose and glucuronate interconversions

*p < 0.05, **p < 0.01

### Correlation analysis between metabolites and efficacy

**Fig.7:**
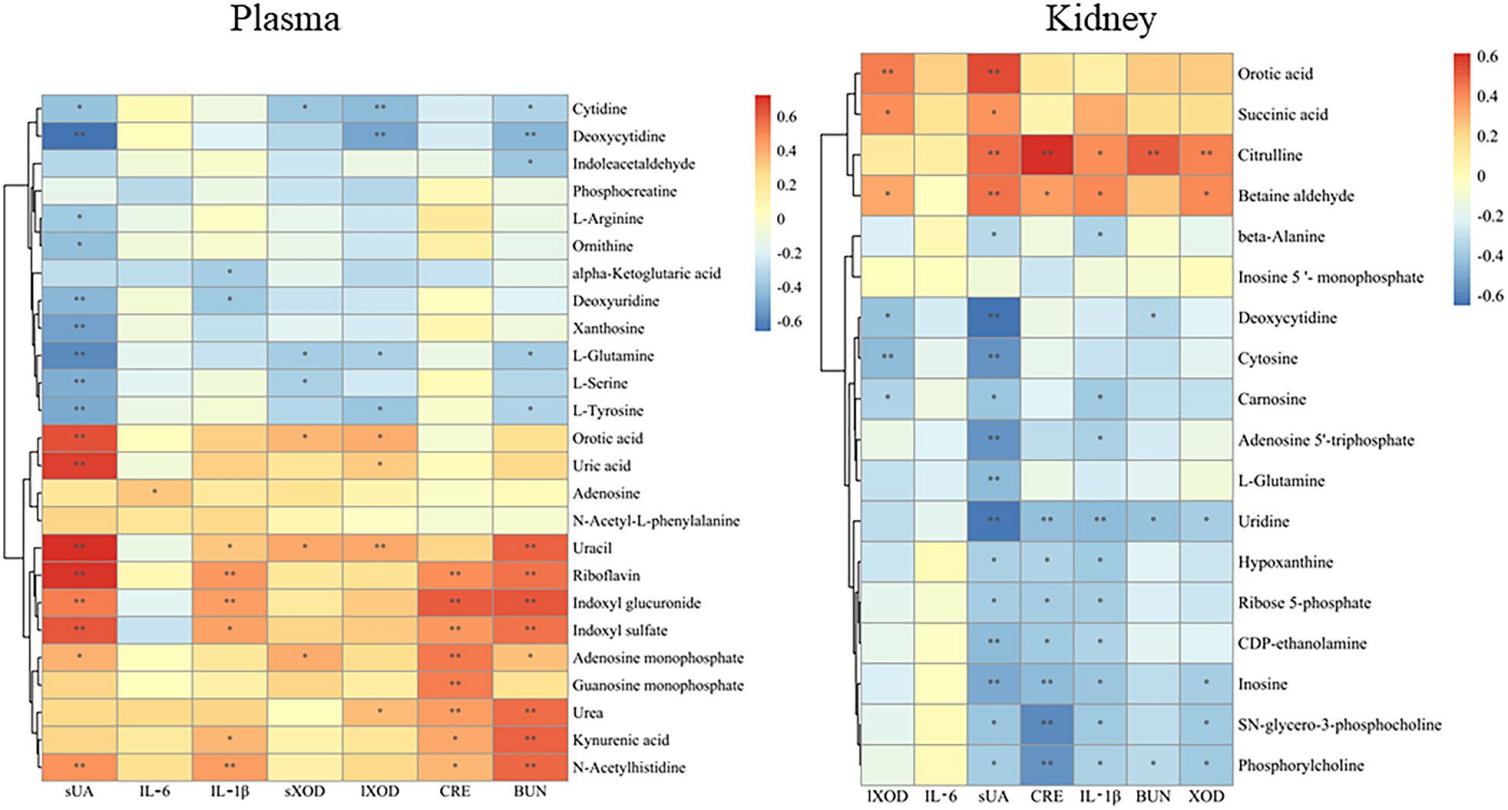
Pearson correlation analysis of differential metabolites and anti-hyperuricemia efficacy. *p < 0.05, **p < 0.01

The correlation analysis was conducted between 43 differential metabolites and the anti-hyperuricemia efficacy of CF and four fractions to clarify the key endogenous metabolites regulated by CF. As shown in Fig. 7, **s**UA was negatively associated with deoxycytidine in plasma (− 0.8 < r < − 0.6, *p* < 0.01). Moreover, it was significantly positively correlated with indoxyl sulfate, orotic acid, riboflavin, uracil, and uric acid (0.8 > r > 0.6, *p* < 0.01). CRE and BUN were positively correlated with indoxyl glucuronide in plasma (0.8 > r > 0.6, *p* < 0.01). **s**UA was negatively correlated with uridine and deoxycytidine in the kidney tissue (− 0.8 < r < − 0.6, *p* < 0.01). CRE was significantly positively associated with citrulline in kidney tissue (0.8 > r > 0.6, *p* < 0.01). These correlations between efficacy and metabolites provide a better understanding of the intervention mechanisms of TFTS. Considering the potential metabolites and the correlation analysis results, riboflavin, uric acid, indoxyl glucuronide, uridine, and citrulline could be necessary biomarkers among the potential metabolites of TFTS treatment against hyperuricemia.

### Metabolic pathway analysis

**Fig.8:**
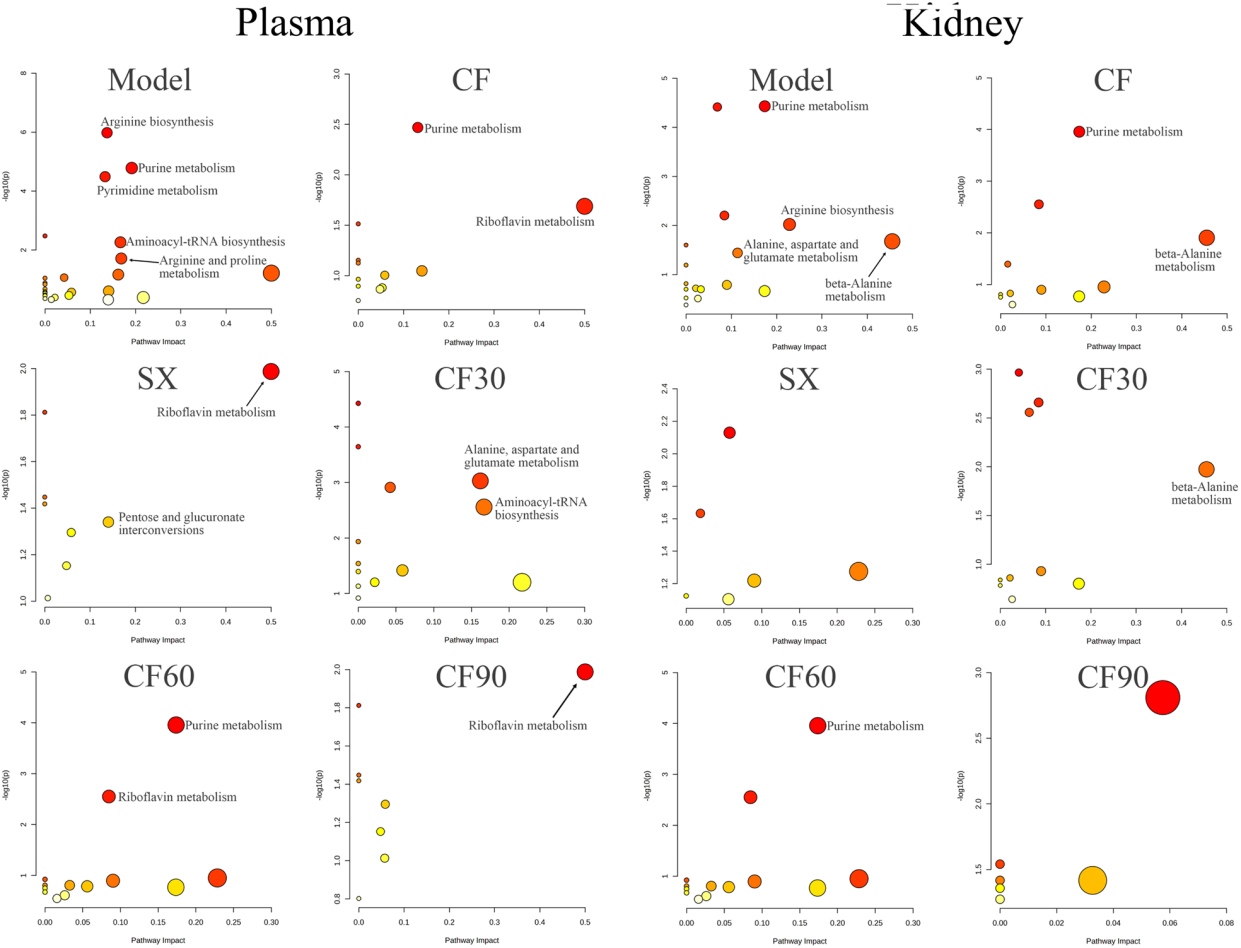
Metabolic pathway analysis of significantly altered metabolites in plasma and kidney

The 25 differential metabolites in plasma and 18 identified metabolites in the kidney were imported into the MetaboAnalyst 5.0 software for pathway analysis. The potential relative metabolic pathways were selected depending on the impact value > 0.1 and *p* < 0.05. The results indicated that these metabolites in the plasma of hyperuricemia rats involved in five pathways met the impact conditions > 0.1 and *p* < 0.05. The five potential metabolic pathways involved arginine biosynthesis, pyrimidine metabolism, purine metabolism, aminoacyl tRNA biosynthesis, and arginine and proline metabolism. Plasma metabonomics indicated that CF was involved in purine and riboflavin metabolism. Moreover, SX was involved in riboflavin metabolism, pentose and glucuronate interconversions metabolism (impact > 0.1 and *p* < 0.05). Additionally, CF30 was associated with alanine, aspartate, glutamate metabolism, and aminoacyl-tRNA biosynthesis (impact > 0.1 and *p* < 0.05). CF60 was involved in the purine and riboflavin metabolism pathway (impact > 0.1 and *p* < 0.05). CF90 was involved in riboflavin metabolism (impact > 0.1 and *p* < 0.05). The kidney metabonomics indicated that purine metabolism, arginine biosynthesis, beta-alanine metabolism, and alanine, aspartate and glutamate metabolism were altered in hyperuricemia rats (impact > 0.1 and *p* < 0.05). After CF treatment, purine metabolism and beta-alanine metabolism were significantly reversed (impact > 0.1 and *p* < 0.05). CF60 was involved in purine metabolism, and CF 30 was associated with beta-alanine metabolism (impact > 0.1 and *p* < 0.05). Additionally, the following disturbed metabolites reversed by CF or CF60 are listed in Table 2, including succinic acid, betaine-aldehyde, SN-glycero-3-phosphocholine, CDP-ethanolamine, Phosphorylcholine, N-Acetylhistidine, kynurenic acid, and indoleacetaldehyde. However, the metabolic pathways of these metabolites were not affected. In general, plasma and kidney metabonomics demonstrated the intervention effect of CF and the four fractions involved different pathways, with detailed results represented in Fig. 8 and Table 2. According to the above plasma and kidney metabonomics result, purine metabolism was the overlapping metabolic pathway after the intervention of CF and CF60, indicating its potential as the anti-hyperuricemia mechanism of TFTS.

## Discussion

We first investigated the anti-hyperuricemia effect of CF and four fractions in the manuscript. The results indicated that CF and CF60 could significantly down-regulate the levels of sUA, CRE, sXOD, lXOD, and IL-1β in hyperuricemia rats. SX, CF30 and CF90 could significantly decrease XOD and IL-1β, or CRE levels in hyperuricemia rats but did not affect uric acid. The results indicated anti-hyperuricemia effect of the four fractions was different, and CF60 was the main CF fraction. In addition, the difference in anti-hyperuricemia efficacy among CF and four fractions remains unclear. Therefore, the metabolite differences between the model and the control groups were compared depending on UPLC-Q-TOF–MS technology combined with multivariate statistical analysis to explore the potential anti-hyperuricemia mechanism of CF and the four fractions.

In hyperuricemia rats, abnormalities were observed in seven metabolic pathways, including purine metabolism, arginine biosynthesis, pyrimidine metabolism, aminoacyl tRNA biosynthesis, arginine and proline metabolism, beta-alanine metabolism, alanine, aspartate, and glutamate metabolism using kidney and plasma metabonomics. The kidney and plasma metabonomics indicated that arginine biosynthesis and purine metabolism were the overlapping disturbed metabolic pathway among the hyperuricemia rats. Plasma and kidney metabonomics demonstrated the purine metabolism was reversed after CF intervention, which indicated that CF exerted an anti-hyperuricemia effect via the purine metabolism signaling pathway. The four CF fractions were involved in differential metabolic pathways due to the proportion and composition of components, and only CF60 could regulate purine metabolism. Thus, CF60 could be the main effective fraction. In addition, the riboflavin metabolic pathway did not change significantly in hyperuricemia rats (*p* > 0.05, impact > 0.1). However, the riboflavin metabolic pathway was affected after the CF and CF60 intervention. Riboflavin is a metabolite of the riboflavin metabolic pathway and behaves as the coenzyme of XOD [22], the critical enzyme involved in purine nucleotide degradation. Thus, the significant elevation of riboflavin in hyperuricemia rats could be associated with increased XOD activity. Consequently, riboflavin metabolism played an auxiliary role in purine metabolism without confirmation.

Purines are essential molecules with multiple functions to maintain the normal physiological function of cells and are involved in the nucleic acid synthesis and energy-requiring reactions. Its metabolism is divided into three parts: biosynthesis, catabolism to uric acid, and salvaging to recover the purine bases provided by the diet or catabolism [23, 24]. Uric acid is the end product of purine metabolism, and elevated level of uric acid indicates a purine metabolic disorder among humans [25]. Uric acid can be degraded to allantoin and urea by uricase in rats [26]. Serum urate concentrations are dependent on the balance between urate production and elimination. A lack of balance causes excessive serum uric acid accumulation, leading to hyperuricemia [25]. Gout is a systemic disease resulting from urate deposition in joints due to uric acid overproduction [27]. The present study observed a higher plasma uric acid level in hyperuricemia rats. Moreover, 12 metabolites attributed to purine metabolism were disturbed in plasma and kidney tissue, including hypoxanthine, inosine 5'-monophosphate (IMP), inosine, adenosine 5'-triphosphate (ATP), L-Glutamine, ribose 5-phosphate, GMP, xanthosine, uric acid, adenosine,urea and AMP [28]. Therefore, hyperuricemia or gout is characterized by purine metabolism disorder. Purine nucleotides are synthesized based on ribose 5-phosphate and ATP. Purine nucleotides interconverted through the enzyme action due to the feedback control, including IMP, GMP, AMP, and ATP. Most of the AMP formed is used for ATP synthesis. However, excessive AMP could promote uric acid synthesis through the purine nucleotide degradation pathway initiated by nucleotide dephosphorylation [26, 29] and nucleoside formation (adenosine, inosine, and guanosine). Adenosine is converted into purine base hypoxanthine through adenosine deaminase (ADA) and purine nucleoside phosphorylase (PNP) activity. Hypoxanthine, the reaction substrate of xanthine oxidase, is converted to uric acid by xanthine oxidase [30]. Moreover, metabolites in purine metabolism, such as ATP, AMP and GMP, are essential molecules controlling intracellular energy homeostasis and nucleotide synthesis. Clinically, hyperuricemia is the emergence of a cell energy crisis [29]. The decreased ATP and IMP levels in the kidney and the increased AMP level in plasma also established the energy metabolism disorder, conversely confirming the purine metabolism disorder. Phosphocreatine can quickly synthesize ATP as an energy-storage form of creatine [31]. The decreased phosphocreatine level indicated the disturbance of energy metabolism in hyperuricemia rats. Additionally, nicotinic acid participated in purine metabolism and increased urate reabsorption [32]. In the present study, these metabolites were reversed partially or wholly in purine metabolism after CF and CF60 treatment.

The kidney is the main metabolic organ for uric acid excretion, excreting about 70% of uric acid in the body [33]. Thus, kidney metabonomics was investigated. Kidney metabonomics indicated that 18 metabolites were disturbed in hyperuricemia rats. These metabolites detected in the kidney, including hypoxanthine, IMP, inosine, ATP, L-glutamine, and ribose 5-Phosphate, demonstrated purine metabolism disorder. Thus, renal injury is a common complication of hyperuricemia due to uric acid and urate overproduction [34]. HE staining confirmed renal injury within hyperuricemia rats. The present study detected multiple metabolites associated with renal injury in the blood of hyperuricemia rats. This included N-acetyl-L-phenylalanine, indoxyl glucuronide, kynurenic acid, N-acetylhistidine, indoxyl sulfate, L-glutamine and L-serine. The results found that N-acetyl-L-phenylalanine, indoxyl glucuronide, kynurenic acid, N-acetylhistidine, and indoxyl sulfate levels were significantly elevated, and that of L-glutamine and L-serine were significantly decreased in hyperuricemia rats. Uremia-related substances, such as N-acetyl-L-phenylalanine, indoxyl glucuronide, kynurenic acid, N-acetylhistidine, and indoxyl sulfate, were increased in hyperuricemia rats [35–39]. Thus, it indicated renal dysfunction in hyperuricemia rats. An imbalance of amino acid metabolism has been closely associated with chronic kidney disease. L-glutamine and L-serine levels in this study were significantly decreased in hyperuricemia rats. Glutamine acts as the NH_3_ donor in the kidney and maintains renal function. Under the hyperuricemia condition, the excess blood uric acid makes the kidney absorb more L-Glutamine to compensate for excess acid [40, 41]. Additionally, the kidney is the primary site of serine production, and a reduced level of serine was observed in hyperuricemia nephropathy [40]. Therefore, the decreased level of L-glutamine and L-serine further suggested renal injury induced by hyperuricemia due to disturbance of purine metabolism. After the administration of CF and CF60, the metabolites associated with renal injury were reversed to a certain degree. CF significantly reduced the level of indoxyl glucuronide. CF60 significantly down-regulated the indoxyl glucuronide, indoxyl sulfate, N-acetylhistidine and kynurenic acid levels. CF improved N-acetylhistidine, kynurenic acid, N-acetyl-L-phenylalanine, L-glutamine, and L-serine levels in hyperuricemia rats. However, there was no significant difference than in the model group. These reversed metabolites could be related to the improvement of renal function. The improvement of renal histology and the down-regulation of sCRE confirmed the protective effect of CF and CF60 on renal function.

The intestinal tract is also an essential excretory organ of uric acid. Approximately 30% of uric acid in the body is excreted through the intestinal tract and metabolized by gut bacteria [42]. Consequently, long-term hyperuricemia can lead to intestinal inflammation, weakened intestinal barrier function, and an imbalance of intestinal flora [43, 44]. The overproduction of uric acid could change the gut microbiota species. Conversely, the gut microbiota and its metabolites can also reduce blood uric acid by promoting purine catabolism [45]. Moreover, they control gut barrier permeability to alleviate chronic inflammation [46–48]. Indole sulfate and indoxyl glucuronide are derived from the tryptophan metabolism of gut microbiota. Indole sulfate induces intestinal inflammation, oxidative stress, and intestinal barrier damage by mitochondrial autophagy injury mediated through the IRF1-DRP1 axis [49–51]. In the present study, indole sulfate and indoxyl glucuronide were significantly elevated in the blood of hyperuricemia rats, consistent with previous studies [52]. Indoleacetaldehyde is the indole metabolite of tryptophan metabolized by gut microbiota and the aryl hydrocarbon receptor (AhR) ligand. It is stimulated by lamina propria lymphocytes (LPLs) to secret IL-22 and activates the aryl hydrocarbon receptor (AhR) [53–57]. Therefore, it shows a protective and anti-inflammatory effect on the intestinal tract. In the present study, indoleacetaldehyde levels decreased significantly in hyperuricemia rats than in normal rats. After the CF or CF60 intervention, indoxyl glucuronide and indoxyl sulfate levels were significantly reduced, and indoleacetaldehyde levels was elevated.

**Fig.9:**
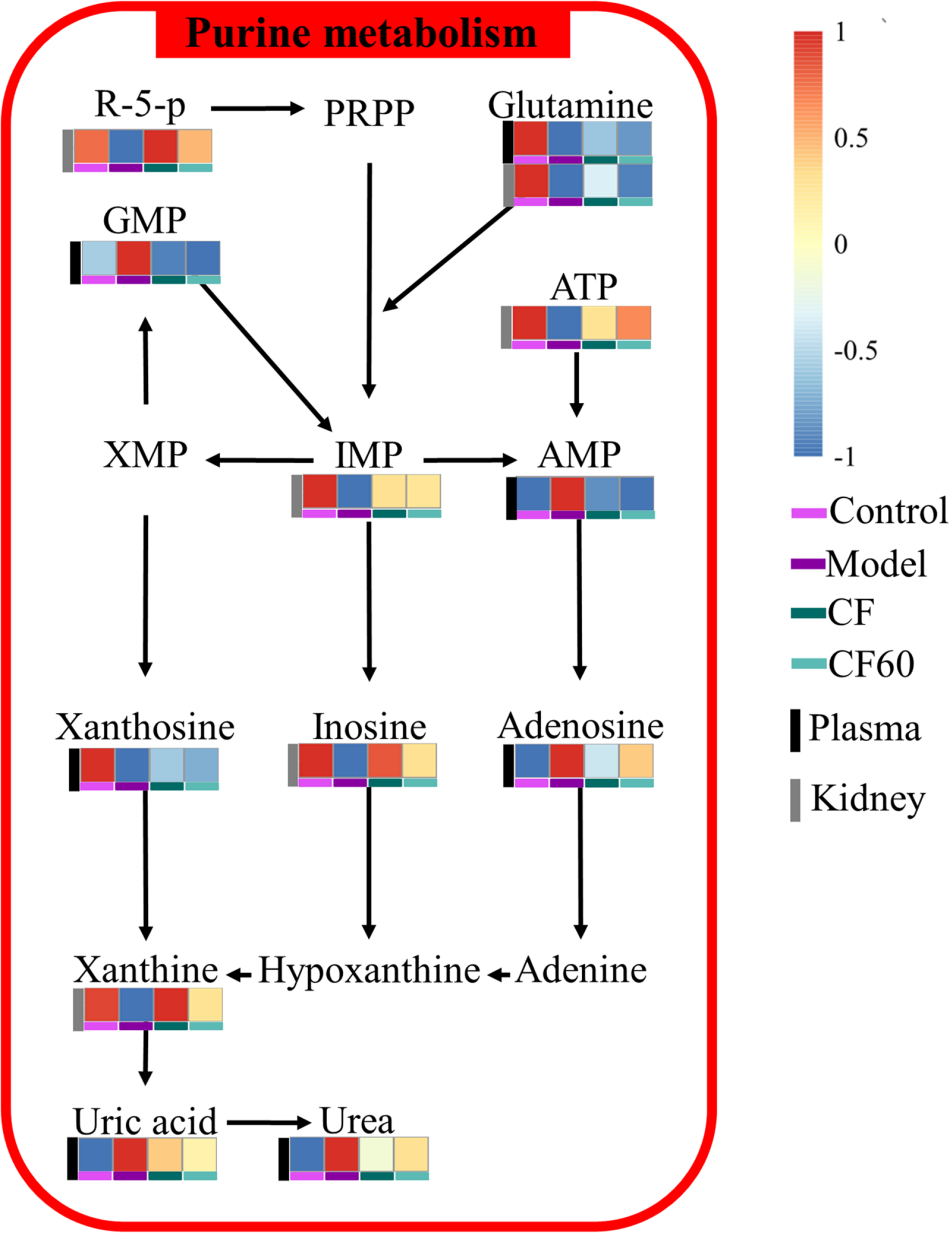
Network of the key biomarkers and pathways according to the KEGG pathway databases

The above results revealed that CF mainly treats hyperuricemia by improving purine metabolism. The metabolic pathways regulated by CF60 were similar to CF. CF60 had significant uric acid-lowering effects, while other fractions had no significant uric acid-lowering effects. Therefore, CF60 could be the main active component of TFTS, and CF and CF60 primarily treat hyperuricemia by improving purine metabolism. The endogenous metabolite changes were constructed based on the KEGG pathway database to clarify the relationship between differential metabolites. The detailed result is depicted in Fig. 9.

## Conclusions

In the present study, biochemical index and histopathology indicated the potential anti-hyperuricemia effect. Forty-three biomarkers and seven disturbed metabolic pathways were obtained using UPLC-MS/MS-based plasma and kidney metabolomics. TFTS could effectively reverse 19 metabolites and alleviate the symptoms of hyperuricemia rats via purine metabolism. Further experiment validation could confirm the potential improvement of intestinal and renal functions. In addition, our findings further confirmed that CF60 was the main active fraction of TFTS. Therefore, the results could provide a reference for the clinical application and promotion of TFTS.

## Supplementary Information

**Additional file 1**: BPI chromatograms of CF and four fractions in negative mode.

**Additional file 2**: The chemical constituents of four fractions by UPLC–ESI-Q-TOF-MS/MS.

**Additional file 3**: The peak area deviation of internal standard in negative and positive ion mode.

**Additional file 4**: Line plots of QC samples of plasma and kidney using PCA mode based on UPLC-MS/MS data

## Data Availability

The datasets during and/or analysed during the current study available from the corresponding author on reasonable request.

## References

[1] Dalbeth N, Merriman TR, Stamp LK (2016). Gout. Lancet.

[2] Zhang S, Wang Y, Cheng J, Huangfu N, Zhao R, Xu Z, Zhang F, Zheng W, Zhang D (2019). Hyperuricemia and cardiovascular disease. Curr Pharm Des.

[3] Benn CL, Dua P, Gurrell R, Loudon P, Pike A, Storer RI, Vangjeli C (2018). Physiology of hyperuricemia and urate-lowering treatments. Front Med.

[4] Li L, Zhang Y, Zeng C (2020). Update on the epidemiology, genetics, and therapeutic options of hyperuricemia. Am J Transl Res.

[5] Paul BJ, Anoopkumar K, Krishnan V (2017). Asymptomatic hyperuricemia: is it time to intervene?. Clin Rheumatol.

[6] Yip K, Cohen RE, Pillinger MH (2020). Asymptomatic hyperuricemia: is it really asymptomatic?. Curr Opin Rheumatol.

[7] Strilchuk L, Fogacci F, Cicero AF (2019). Safety and tolerability of available urate-lowering drugs: a critical review. Expert Opin Drug Saf.

[8] Day RO, Kannangara DR, Stocker SL, Carland JE, Williams KM, Graham GG (2017). Allopurinol: insights from studies of dose-response relationships. Expert Opin Drug Metab Toxicol.

[9] Tibet Health Bureau (1979). Tibetan Medicine Standard.

[10] Li D L, Xu C L, Yang S S, Cheng B S, D Z Z. Great Dictionary of Chinese Medicine. Beijing: China Pharmaceutical Science and Technology Press; 1991.

[11] Dajie JC (2018). Analysis on the clinical effective rate of Tibetan medicine Tongfeng Decoction in the treatment of zhinai (gout). Health Guide.

[12] Nanjie XW, Cairang ZM (2017). Clinical observation of Tibetan medicine Tongfeng Decoction in the treatment of zhinai (gout). Chin J Ethnomed Ethnopharm.

[13] Chen HF, Zhang C, Yao Y, Li JM, Du WD, Li ML, Wu B, Yang SL, Feng YL, Zhang WG (2019). Study on anti-hyperuricemia effects and active ingredients of traditional Tibetan medicine TongFengTangSan (TFTS) by ultra-high-performance liquid chromatography coupled with quadrupole time-of-flight mass spectrometry. J Pharm Biomed Anal.

[14] Zhang XR, Qiao YJ, Zhu HT, Kong QH, Wang D, Yang CR, Zhang YJ (2021). Multiple in vitro biological effects of phenolic compounds from *Terminalia* chebula var tomentella. J Ethnopharmacol.

[15] Dhingra AK, Chopra B, Grewal AS, Guarve K (2022). Pharmacological properties of Chebulinic acid and related ellagitannins from nature: an emerging contemporary bioactive entity. Pharmacol Res Modern Chinese Med.

[16] Ishimoto H, Shibata M, Myojin Y, Ito H, Sugimoto Y, Tai A, Hatano T (2011). *In vivo* anti-inflammatory and antioxidant properties of ellagitannin metabolite urolithin A. Bioorg Med Chem Lett.

[17] Pham AT, Malterud KE, Paulsen BS, Diallo D, Wangensteen H (2011). DPPH Radical scavenging and xanthine oxidase inhibitory activity of *Terminalia macroptera* Leaves. Nat Prod Commun.

[18] Wang Y, Zhu W, Lu D, Zhang C, Wang Y (2021). Tetrahydropalmatine attenuates MSU crystal-induced gouty arthritis by inhibiting ROS-mediated NLRP3 inflammasome activation. Int Immunopharmacol.

[19] Bujak R, Struck-Lewicka W, Markuszewski MJ, Kaliszan R (2015). Metabolomics for laboratory diagnostics. J Pharm Biomed Anal.

[20] Cañadas-Garre M, Anderson K, McGoldrick J, Maxwell AP, McKnight AJ (2019). Proteomic and metabolomic approaches in the search for biomarkers in chronic kidney disease. J Proteomics.

[21] Schrimpe-Rutledge AC, Codreanu SG, Sherrod SD, McLean JA (2016). Untargeted metabolomics strategies-challenges and emerging directions. J Am Soc Mass Spectrom.

[22] Naghashpour M, Jafarirad S, Amani R, Sarkaki A, Saedisomeolia A (2017). Update on riboflavin and multiple sclerosis: a systematic review. Iran J Basic Med Sci.

[23] Braun-Falco O, Plewig G, Wolff HH, Burgdorf WH (2000). Disorders of Purine Metabolism Dermatology.

[24] Dewulf JP, Marie S, Nassogne MC (2021). Disorders of purine biosynthesis metabolism. Mol Genet Metab.

[25] Keenan RT, Pillinger MH (2013). Etiology and pathogenesis of hyperuricemia and gout. Rheumatology.

[26] Jiménez RT, Puig JG (2012). Purine metabolism in the pathogenesis of hyperuricemia and inborn errors of purine metabolism associated with disease, Gout & Other Crystal Arthropathies.

[27] Ragab G, Elshahaly M, Bardin T (2017). Gout: an old disease in new perspective–a review. J Adv Res.

[28] Blanco A, Blanco G (2022). Chapter 18 Purine and pyrimidine metabolism Medical Biochemistry 2nd.

[29] Fox IH, Palella TD, Kelley WN (1987). Hyperuricemia: a marker for cell energy crisis. New England J Med.

[30] Schmidt HM, Kelley EE, Straub AC (2019). The impact of xanthine oxidase (XO) on hemolytic diseases. Redox Biol.

[31] Dickinson H, Bain E, Wilkinson D, Middleton P, Crowther CA, Walker DW (2014). Creatine for women in pregnancy for neuroprotection of the fetus. Cochrane Database Syst Rev.

[32] Ben Salem C, Slim R, Fathallah N, Hmouda HJR (2017). Drug-induced hyperuricaemia and gout. Rheumatology.

[33] Yanai H, Adachi H, Hakoshima M, Katsuyama H (2021). Molecular biological and clinical understanding of the pathophysiology and treatments of hyperuricemia and its association with metabolic syndrome, cardiovascular diseases and chronic kidney disease. Int J Mol Sci..

[34] Johnson RJ, Bakris GL, Borghi C, Chonchol MB, Feldman D, Lanaspa MA, Merriman TR, Moe OW, Mount DB, Sanchez Lozada LG, Stahl E, Weiner DE, Chertow GM (2018). Hyperuricemia, acute and chronic kidney disease, hypertension, and cardiovascular disease: report of a scientific workshop organized by the national kidney foundation. Am J Kidney Dis.

[35] Etinger A, Kumar SR, Ackley W, Soiefer L, Chun J, Singh P, Grossman E, Matalon A, Holzman RS, Meijers B, Lowenstein J (2018). The effect of isohydric hemodialysis on the binding and removal of uremic retention solutes. PLoS ONE.

[36] Lano G, Burtey S, Sallée M (2020). Indoxyl sulfate, a uremic endotheliotoxin. Toxins.

[37] Mair RD, Sirich TL, Meyer TW (2018). Uremic toxin clearance and cardiovascular toxicities. Toxins.

[38] Tanaka H, Sirich TL, Plummer NS, Weaver DS, Meyer TW (2015). An enlarged profile of uremic solutes. PLoS ONE.

[39] Vanholder R, Pletinck A, Schepers E, Glorieux G (2018). Biochemical and clinical impact of organic uremic retention solutes: a comprehensive update. Toxins.

[40] Han B, Gong M, Li Z, Qiu Y, Zou Z (2020). NMR-based metabonomic study reveals intervention effects of polydatin on potassium oxonate-induced hyperuricemia in rats. Oxid Med Cell Longev.

[41] Pan L, Han P, Ma S, Peng R, Wang C, Kong W, Cong L, Fu J, Zhang Z, Yu H, Wang Y, Jiang J (2020). Abnormal metabolism of gut microbiota reveals the possible molecular mechanism of nephropathy induced by hyperuricemia. Acta Pharm Sin B.

[42] Yun Y, Yin H, Gao Z, Li Y, Gao T, Duan J, Yang R, Dong X, Zhang L, Duan W (2017). Intestinal tract is an important organ for lowering serum uric acid in rats. PLoS ONE.

[43] Lv Q, Xu D, Zhang X, Yang X, Zhao P, Cui X, Liu X, Yang W, Yang G, Xing S (2020). Association of hyperuricemia with immune disorders and intestinal barrier dysfunction. Front Physiol.

[44] Zhao H, Lu Z, Lu Y (2022). The potential of probiotics in the amelioration of hyperuricemia. Food Funct.

[45] Wang J, Chen Y, Zhong H, Chen F, Regenstein J, Hu X, Cai L, Feng F (2021). The gut microbiota as a target to control hyperuricemia pathogenesis: potential mechanisms and therapeutic strategies. Crit Rev Food Sci Nutr.

[46] Brito JS, Borges NA, Esgalhado M, Magliano DC, Soulage CO, Mafra D (2017). Aryl hydrocarbon receptor activation in chronic kidney disease: role of uremic toxins. Nephron.

[47] King LJ, Parke DV, Williams RT (1966). The metabolism of [2-14C] indole in the rat. Biochem J.

[48] Niwa T, Miyazaki T, Tsukushi S, Maeda K, Tsubakihara Y, Owada A, Shiigai T (1996). Accumulation of indoxyl-beta-D-glucuronide in uremic serum: suppression of its production by oral sorbent and efficient removal by hemodialysis. Nephron.

[49] Adesso S, Ruocco M, Rapa SF, Piaz FD, Raffaele Di Iorio B, Popolo A, Autore G, Nishijima F, Pinto A, Marzocco S (2019). Effect of indoxyl sulfate on the repair and intactness of intestinal epithelial cells: role of reactive oxygen species’ release. Int J Mol. Sci..

[50] Huang Y, Zhou J, Wang S, Xiong J, Chen Y, Liu Y, Xiao T, Li Y, He T, Li Y, Bi X, Yang K, Han W, Qiao Y, Yu Y, Zhao J (2020). Indoxyl sulfate induces intestinal barrier injury through IRF1-DRP1 axis-mediated mitophagy impairment. Theranostics.

[51] Rapa SF, Prisco F, Popolo A, Iovane V, Autore G, Di Iorio BR, Dal Piaz F, Paciello O, Nishijima F, Marzocco S (2021). Pro-Inflammatory effects of indoxyl sulfate in mice: impairment of intestinal homeostasis and immune response. Int J Mol Sci.

[52] Guo Y, Yu Y, Li H, Ding X, Li X, Jing X, Chen J, Liu G, Lin Y, Jiang C, Liu Z, He Y, Li C, Tian Z (2021). Inulin supplementation ameliorates hyperuricemia and modulates gut microbiota in Uox-knockout mice. Eur J Nutr.

[53] Hou Q, Ye L, Liu H, Huang L, Yang Q, Turner JR, Yu Q (2018). Lactobacillus accelerates ISCs regeneration to protect the integrity of intestinal mucosa through activation of STAT3 signaling pathway induced by LPLs secretion of IL-22. Cell Death Differ.

[54] Lavelle A, Sokol H (2020). Gut microbiota-derived metabolites as key actors in inflammatory bowel disease. Nat Rev Gastroenterol Hepatol.

[55] Puccetti M, Pariano M, Borghi M, Barola C, Moretti S, Galarini R, Mosci P, Ricci M, Costantini C, Giovagnoli S (2021). Enteric formulated indole-3-carboxaldehyde targets the aryl hydrocarbon receptor for protection in a murine model of metabolic syndrome. Int J Pharm.

[56] Zelante T, Iannitti RG, Cunha C, De Luca A, Giovannini G, Pieraccini G, Zecchi R, D'Angelo C, Massi-Benedetti C, Fallarino F, Carvalho A, Puccetti P, Romani L (2013). Tryptophan catabolites from microbiota engage aryl hydrocarbon receptor and balance mucosal reactivity via interleukin-22. Immunity.

[57] Zelante T, Puccetti M, Giovagnoli S, Romani L (2021). Regulation of host physiology and immunity by microbial indole-3-aldehyde. Curr Opin Immunol.

