# Supplementary figures and images for: Exploration of the anti-hyperuricemia effect of TongFengTangSan (TFTS) by UPLC-Q-TOF/MS-based non-targeted metabonomics
Source: Chin Med. 2023 Feb 16;18:17. doi: 10.1186/s13020-023-00716-w (PMC9933412; doi:10.1186/s13020-023-00716-w)

**
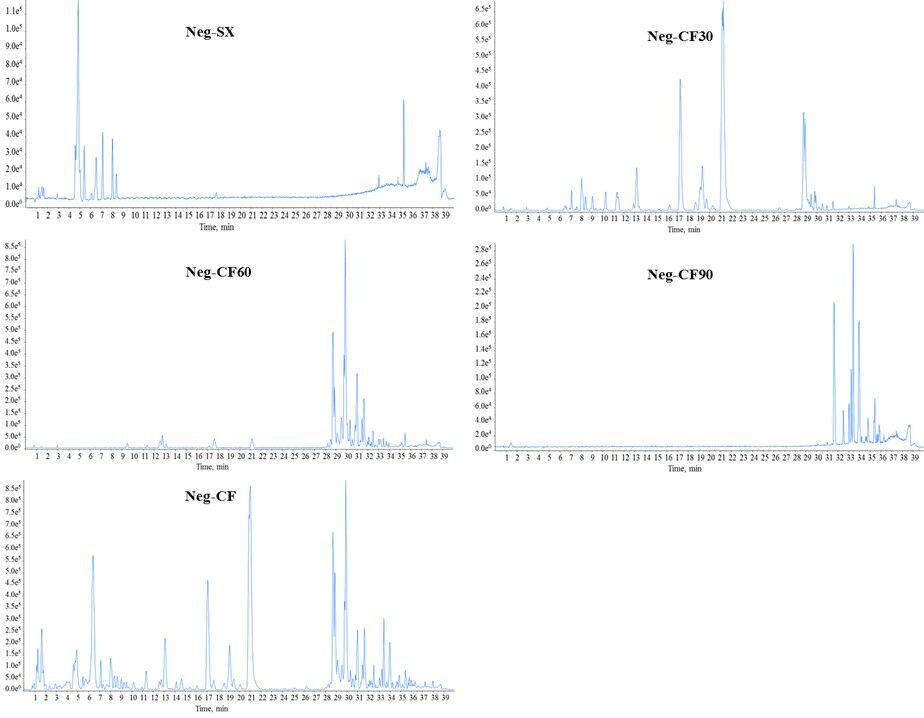
**

Additional file 1 BPI chromatograms of CF and four fractions in negative mode

Supplement: Supplementary file 1 — Additional file 1: BPI chromatograms of CF and four fractions in negative mode. [file 13020_2023_716_MOESM1_ESM.docx]
