# Supplementary material for: Exploration of the anti-hyperuricemia effect of TongFengTangSan (TFTS) by UPLC-Q-TOF/MS-based non-targeted metabonomics
Source: Chin Med. 2023 Feb 16;18:17. doi: 10.1186/s13020-023-00716-w (PMC9933412; doi:10.1186/s13020-023-00716-w)
Supplement: Supplementary file 2 — Additional file 2: The chemical constituents of four fractions by UPLC–ESI-Q-TOF-MS/MS. [file 13020_2023_716_MOESM2_ESM.docx]

**Additional file 2 The chemical constituents of four fractions by UPLC–ESI-Q-TOF-MS/MS**

| Sample | Metabolite name | Average Rt(min) | Average  Mz | Formula | Adduct  type | Reference | ppm | MS^2^ |
| --- | --- | --- | --- | --- | --- | --- | --- | --- |
| SX | Shikimic Acid | 1.74 | 173.0460 | C_7_H_10_O_5_ | [M-H]^-^ | 173.0456 | 2.8 | 173.0512,137.0279,111.0472,93.0404,83.0559,73.0366 |
|  | Uric acid | 2.23 | 167.0216 | C_5_H_4_N_4_O_3_ | [M-H]^-^ | 167.0211 | 3.2 | 167.0289,124.0201,96.0257,69.0165 |
|  | Gallic acid | 4.57 | 169.0143 | C_7_H_6_O_5_ | [M-H]^-^ | 169.0143 | 0.8 | 169.0158,125.0275,125.0275124.0187,107.0175,97.0334,  79.0236,69.0416,51.0321 |
| CF30 | Hippuric acid | 11.24 | 178.0517 | C_9_H_9_NO_3_ | [M-H]^-^ | 178.0510 | 4.2 | 178.0524,134.0669,77.0465 |
|  | Corilagin | 17.25 | 633.0733 | C_27_H_22_O_18_ | [M-H]^-^ | 633.0733 | -0.1 | 633.0692,463.0509,300.9999,275.0194 |
|  | 1,3,6-tri-O-galloylglucose | 21.26 | 635.0903 | C_27_H_24_O_18_ | [M-H]^-^ | 635.0890 | 2.2 | 635.0822,635.0822483.0731,465.0617,313.0557,221.0459,  169.0161,125.0261 |
|  | 1,2,3,6-tetragalloylglucose | 28.92 | 787.0984 | C_34_H_28_O_22_ | [M-H]^-^ | 787.1000 | -1.9 | 787.0928,635.0818,617.0751,465.0634,295.0427,169.0139 |
|  | Chebulagic acid | 28.76 | 953.0855 | C_41_H_30_O_27_ | [M-H]^-^ | 953.0902 | -4.8 | 953.0716,783.0662,633.0651,481.0566,300.9961,275.018 |
| CF60 | Magnolflorine | 9.52 | 340.1551 | C_20_H_23_NO_4_ | [M-H]^-^ | 340.1554 | -1 | 340.1585,325.1304,310.1064,282.1139,252.0418,224.0481 |
|  | Salicylic acid or isomer | 12.49 | 137.0250 | C_7_H_6_O_3_ | [M-H]^-^ | 137.0244 | 4.9 | 137.032,93.0411,65.0487 |
|  | Ethyl gallate | 17.63 | 197.0450 | C_9_H_10_O_5_ | [M-H]^-^ | 197.0456 | -2.8 | 197.0459,169.014,124.0186 |
|  | Chebulagic acid | 28.76 | 953.0855 | C_41_H_30_O_27_ | [M-H]^-^ | 953.0902 | -4.8 | 953.0716,783.0662,633.0651,481.0566,300.9961,275.018 |
|  | Ellagic Acid | 29.20 | 301.0000 | C_14_H_6_O_8_ | [M-H]^-^ | 300.9990 | 3.6 | 283.9978,254.0096,254.0096245.0076,229.0128,201.0212,  185.0265,173.0271,145.0327 |
|  | Beta-penta-O-galloyl-glucose | 29.80 | 939.1150 | C_41_H_32_O_26_ | [M-H]^-^ | 939.1109 | 4.4 | 939.1044,769.0875,617.0887,447.0535,169.0131 |
|  | Chebulinic acid | 29.81 | 955.1019 | C_41_H_32_O_27_ | [M-H]^-^ | 955.1058 | -4.1 | 955.0916,785.0793,337.0165,319.0062,283.0315,275.018 |
|  | Feruloyltyramine | 31.42 | 312.1243 | C_18_H_19_NO_4_ | [M-H]^-^ | 312.1241 | 0.7 | 312.1217,312.1217297.0986,253.0913,190.0524,178.054,  148.0552,135.05 |
| CF90 | Madecassic acid | 33.86 | 503.3365 | C_30_H_48_O_6_ | [M-H]^-^ | 503.3378 | -2.5 | 485.3252,453.2993,441.3358,409.3047 |
|  | Formononetine | 33.65 | 267.0673 | C_16_H_12_O_4_ | [M-H]^-^ | 267.0663 | 3.9 | 267.0664,252.0428,223.0412,195.0427,167.0551,132.0221 |
|  | Rhein | 35.30 | 283.0242 | C_15_H_8_O_6_ | [M-H]^-^ | 283.0248 | -2.0 | 283.0255,239.0346,211.0418,183.0493 |
