# Supplementary material for: Exploration of the anti-hyperuricemia effect of TongFengTangSan (TFTS) by UPLC-Q-TOF/MS-based non-targeted metabonomics
Source: Chin Med. 2023 Feb 16;18:17. doi: 10.1186/s13020-023-00716-w (PMC9933412; doi:10.1186/s13020-023-00716-w)
Supplement: Supplementary file 3 — Additional file 3: The peak area deviation of internal standard in negative and positive ion mode. [file 13020_2023_716_MOESM3_ESM.docx]

| **Additional file 3. The peak area deviation of internal standard in negative and positive ion mode** | | | | | | |
| --- | --- | --- | --- | --- | --- | --- |
| **Source** | **Negative Mode** | | | **Positive Mode** | | |
|  | **m/z** | **Average Rt (min)** | **Area (RSD %)** | **m/z** | **Average Rt (min)** | **Area (RSD %)** |
| **Plasma** | 133.1061 | 247.698 | 2.32% | 135.1202 | 247.17 | 1.47% |
| **Kidney** | 133.1062 | 247.091 | 4.46% | 135.1199 | 247.969 | 3.23% |
