# Supplementary material for: Exploration of the anti-hyperuricemia effect of TongFengTangSan (TFTS) by UPLC-Q-TOF/MS-based non-targeted metabonomics
Source: Chin Med. 2023 Feb 16;18:17. doi: 10.1186/s13020-023-00716-w (PMC9933412; doi:10.1186/s13020-023-00716-w)
Supplement: Supplementary file 4 — Additional file 4: Line plots of QC samples of plasma and kidney using PCA mode based on UPLC-MS/MS data [file 13020_2023_716_MOESM4_ESM.docx]

**
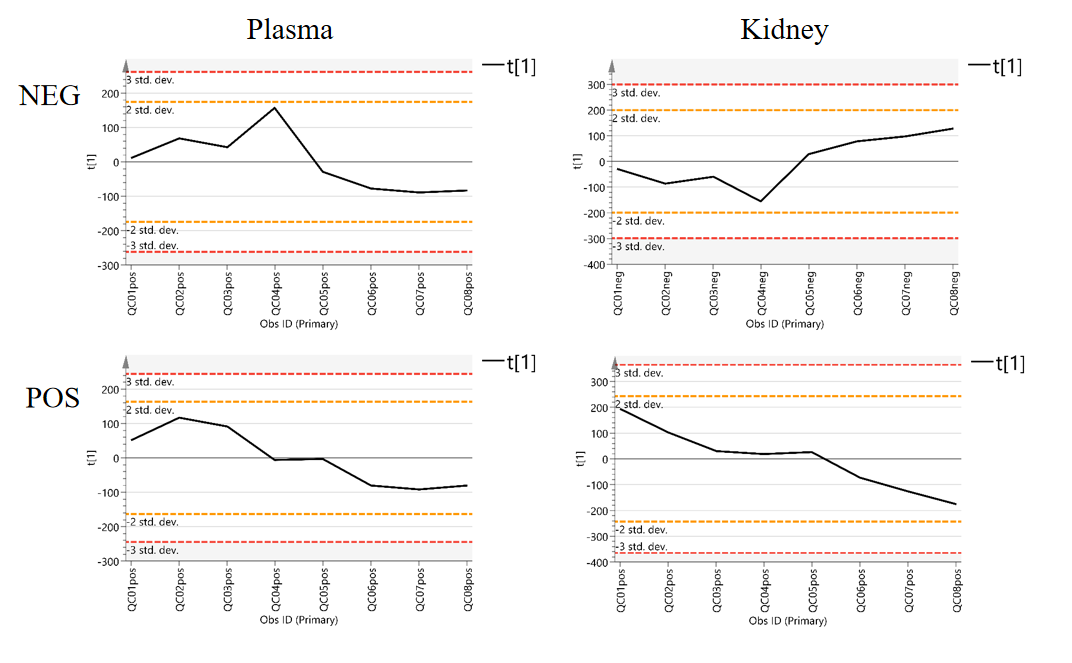
**

**Additional file 4.** Line plots of QC samples of plasma and kidney using PCA mode based on UPLC-MS/MS data.
